# Transient Antiskyrmion‐Mediated Topological Transitions in Isotropic Magnets

**DOI:** 10.1002/advs.202513126

**Published:** 2026-01-04

**Authors:** Bingqian Dai, Tianyi Wang, Albert Lee, Shijie Xu, Zhongjian Bian, Xinyue Zhu, Puyang Huang, Aadi Chaturvedi, Yaochen Li, Yang Cheng, Qingyuan Shu, Haoran He, Hanshen Huang, Lixuan Tai, Kin Wong, Jinbo Yang, Anjan Soumyanarayanan, Zhaochu Luo, Kang L. Wang

**Affiliations:** ^1^ Department of Electrical and Computer Engineering University of California Los Angeles CA 90095 USA; ^2^ State Key Laboratory of Artificial Microstructure and Mesoscopic Physics Institute of Condensed Matter Physics and Materials School of Physics Peking University Beijing 100871 China; ^3^ ICY Technology (Beijing) Co., Ltd Beijing 100871 China; ^4^ Department of Physics National University of Singapore Singapore 117551 Singapore

**Keywords:** magnetic devices, skyrmionic materials, spintronics, topological spin textures, unconventional computing

## Abstract

From elementary particles to cosmological structures, topological solitons are ubiquitous nonlinear excitations valued for their robustness and complex interactions. In magnetism, solitons such as skyrmions and antiskyrmions behave analogously to particles and antiparticles, typically annihilating in pairs in accordance with topological conservation laws. Here the stripe‐to‐skyrmion transition is experimentally observed and a model for a skyrmion–antiskyrmion–skyrmion intertwined state is introduced, in which the central antiskyrmion is annihilated, leading to an increase in the local topological number. Because this transition occurs repeatedly across the film, the cumulative effect produces a global increase in the total topological charge. This model reflects a breakdown of topological protection in isotropic Dzyaloshinskii–Moriya interaction (DMI) materials, where symmetry constraints render the antiskyrmion energetically unstable and thermally activated. Using micromagnetic simulations and minimum‐energy‐path calculations, the antiskyrmion is identified as a transient, metastable excitation. To highlight its functional potential, this stripe‐to‐skyrmion transition within a Hall device is exploited to generate stochastic bitstreams, which are subsequently used in a proof‐of‐concept probabilistic computing demonstration. These results contribute to the understanding of topological spin‐texture dynamics and suggest opportunities for leveraging their transient behavior in probabilistic computing architectures.

## Graphical abstract

A transient antiskyrmion‐mediated pathway that drives repeated stripe‐to‐skyrmion transitions is revealed, producing a net increase in topological charge in isotropic Dzyaloshinskii–Moriya interaction films. Experiments and simulations identify the antiskyrmion as a metastable excitation, enabling stochastic bitstream generation for probabilistic computing.

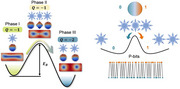

## 1 Introduction

**Figure 1:**
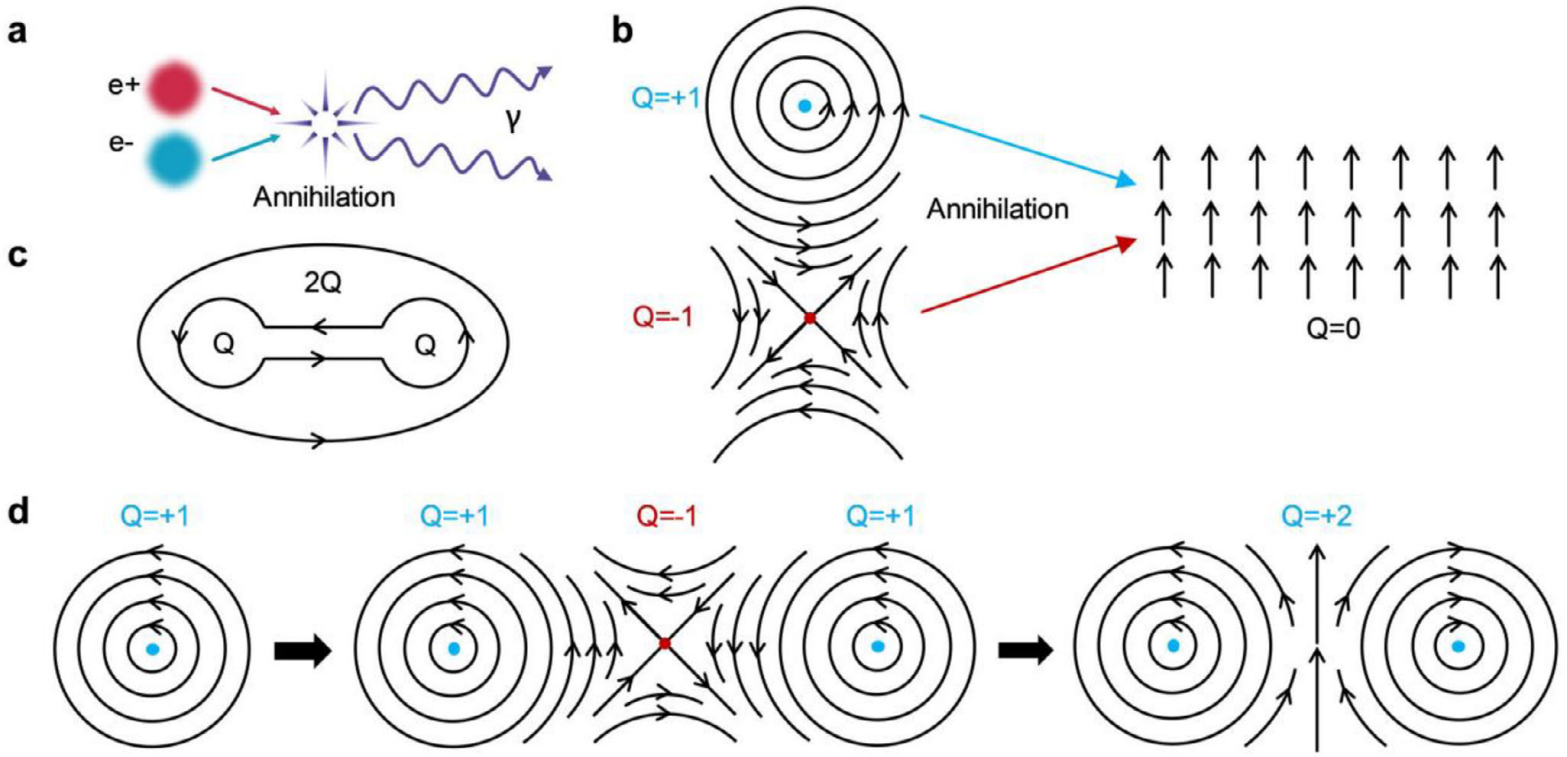
Proposed topological transition based on soliton‐antisoliton interactions. a) Annihilation of an electron and positron, emitting gamma radiation, shown for analogy. b) Coalescence and annihilation of a magnetic soliton (topological number *Q* = +1) and antisoliton (*Q* = −1) into a topologically trivial ferromagnetic state (*Q* = 0), exemplified by a vortex‐antivortex pair. Spin orientations are indicated by black arrows. c) Conservation of global topological number through the enclosing of local solitons. d) Proposed topological transition involving a soliton–antisoliton–soliton intertwined configuration. Left: Initial soliton state (*Q* = +1). Middle: Formation of a soliton‐antisoliton‐soliton intertwined state, conserving *Q* = 1 − 1 + 1 = +1. Right: Annihilation of the central antisoliton driven by symmetry breaking and thermal activation, resulting in a two‐soliton final state with increased topological number (*Q* = +2).

Magnetic topological solitons ^[^ ^1^ ^]^ are continuous, non‐trivial vector field configurations that cannot be smoothly deformed into a uniform state, offering intrinsic topological protection. These textures, characterized by an integer‐valued topological index, exhibit particle‐like behavior and interact through well‐defined topological rules. Analogous to the annihilation processes of elementary particles and antiparticles such as electrons and positrons (**Figure** **1**a),^ [^ ^2^ ^]^ magnetic solitons and antisolitons typically undergo coalescence and annihilate into a uniform ferromagnetic state by rotating their spin configurations (Figure 1b), thereby conserving the total topological number (Figure 1c) .^[^ ^3^ ^]^ This foundational picture has been enriched by a series of elegant studies that have captured soliton–antisoliton behaviors under varied conditions, offering valuable insights into their stability, evolution, and controllability, and laying critical groundwork for exploring nontrivial topological phenomena in magnetic systems.^[^ ^4^, ^5^, ^6^, ^7^, ^8^, ^9^, ^10^, ^11^, ^12^, ^13^, ^14^, ^15^, ^16^, ^17^, ^18^, ^19^, ^20^, ^21^ ^]^

Contrary to this conventional framework, we propose a different soliton transition model. As shown in Figure 1d (left), a magnetic soliton—such as a vortex or skyrmion—can form in an isotropic environment, such as one with *C_nv_* symmetry and isotropic DMI .^[^ ^22^, ^23^ ^]^ This soliton can undergo deformation into a more complex, intertwined soliton–antisoliton–soliton state (Figure 1d, middle), which still conserves the topological number. However, the central antisoliton in this composite state is fundamentally unstable, relying on anisotropic DMI for stability—for example, antiskyrmions require systems with *D*_2*d*_ or *S*_4_ symmetry .^[^ ^24^ ^]^ The isotropic environment breaks this symmetry, reducing the energy barrier protecting the antisoliton and rendering it susceptible to external perturbations. Consequently, under thermal fluctuations, the antisoliton becomes a transient excitation that decays into the ferromagnetic background, while the two neighboring solitons remain stable (Figure 1d, right). Each such local event increases the enclosed topological charge by +1, and the accumulation of these local transitions across the film leads to a global increase in the net topological number.

This model suggests that soliton interactions can proceed through fragmentation and partial annihilation pathways different from conventional conservation‐based frameworks. In addition to its theoretical implications, the phenomenon also suggests potential functionality: the transient antisoliton state exhibits thermally activated stochastic behavior, offering a source of randomness. Building on our fundamental study of soliton dynamics, we further demonstrate how their intrinsic stochasticity can be harnessed to generate bitstreams and perform probabilistic tasks, such as solving optimization problems using an Ising machine .^[^ ^25^, ^26^, ^27^, ^28^ ^]^ In contrast to prior device‐level efforts that operate within single‐domain regimes—such as magnetic tunnel junctions with low‐energy switching thresholds ^[^ ^29^ ^]^—our approach expands the operational landscape to include complex, topologically governed spin textures.

Taken together, our work advances the understanding of soliton interactions under symmetry and thermal constraints and opens new opportunities for topological spin‐texture engineering in functional spintronics.

## 2 Results

### 2.1 Skyrmion Fragmentation and the Transient Antiskyrmions Model

**Figure 2:**
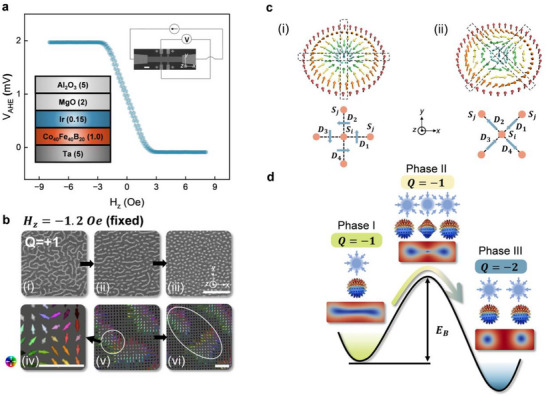
Observation of stripe‐to‐skyrmion transition. a) Hysteresis loop from AHE measurements. Top right: AHE measurement setup, with a DC current *I* = 1 *mA* applied and the transverse voltage *V_AHE_* measured during the out‐of‐plane field *H_z_* sweeping. Scale bar: 20 µ*m*. Middle: AHE Hysteresis loop. Bottom left: Material stack, number in nm. b) MOKE images at − 1.2 *Oe* fixed field and corresponding simulations illustrating spin texture evolution during the transition process. i) Stripe domains evolve into ii) skyrmions, and eventually forming iii) a skyrmion lattice. iv) Simulated antiskyrmion spin texture during the transition, where bright (dark) colors indicate + *M_z_* (− *M_z_*). A color wheel on the left represents in‐plane spin directions. v) Zoomed‐out view of the stripe domain containing the transient antiskyrmion shown in iv), highlighted by a white circle. vi) Antiskyrmion is annihilated by thermal activation, leading to the transformation of the stripe domain into two skyrmions, highlighted by a white oval. Top scale bar: 20 µ*m*; bottom scale bar: 20 *nm*. c) Stabilization of topological textures by DMI. i) Isotropic DMI in *C_nv_* systems favor Néel‐type skyrmions. ii) Anisotropic DMI in *D*_2*d*_ symmetry stabilizes antiskyrmions. d) Schematic of the topological transition involving transient antiskyrmions. Phase I: Initial stripe domain state. From bottom to top: Top view of stripe domain (blue: − *M_z_*, red: + *M_z_*, white: in‐plane spin), mapping to a unit sphere, and a cartoon of the texture (skyrmion core marked by a blue circle and in‐plane spin by the blue arrows), topological number of this phase. Phase II: Skyrmion‐antiskyrmion‐skyrmion intertwined state. One central transient antiskyrmion and two end skyrmions are created. Phase III: Final two‐skyrmion state. Final state following the annihilation of the central antiskyrmion, leaving two stable skyrmions. Phase I and III reside in two potential wells, separated by an energy barrier *E_B_* created by the transient antiskyrmion in Phase II. Energy levels are color‐coded (yellow: highest, green: intermediate, blue: lowest), and the arrow indicates the transition pathway.

In a typical magnetic system with relatively small thermal perturbations, the energetics are predominantly governed by magnetic free energy. During the sweeping of the hysteresis loop, each magnetic state settles into a deep potential well, significantly deeper than thermal energy. After the application of a magnetic field, the magnetic domains quickly reach equilibrium within a microsecond to nanosecond timescale,^[^ ^30^ ^]^ governed by the Landau‐Lifshitz‐Gilbert (LLG) dynamics. Consequently, the magnetic domains remain stable, and the effects of thermal fluctuations are minimal. In the Ta/CoFeB/Ir/MgO heterostructure under investigation (see Experimental Section for material growth details, Supporting Information), the system's energy barrier, i.e., perpendicular magnetic anisotropy (PMA), can be fine‐tuned by the Iridium insertion thickness (Figure S1, Supporting Information). In the low barrier state, indicated by the small saturation field (3 *Oe*) of the anomalous Hall effect (AHE) hysteresis loop shown in **Figure** **2**a, the domain evolution is significantly slower (10^−6^ to 10^−9^ times) than typical domain evolution rates. All of our experiments were carried out under strictly fixed magnetic fields, with no change applied during the observation. When the field was maintained at − 1.2 *Oe*, stripe (labyrinth) domains were initially observed, which progressively divided into skyrmions and eventually formed a skyrmion lattice state over tens of seconds. This transformation is depicted in Figure 2b using Polar Magneto‐Optic Kerr Effect (MOKE) microscopy (refer to Experimental Section for measurement techniques). The initial white stripes (Figure 2b(i))—stripe domains—gradually evolved into white circles (b(ii))—skyrmions—eventually forming a skyrmion lattice (b(iii)). In these images, white and black colors represent positive and negative out‐of‐plane magnetizations (+ *M_z_* and − *M_z_*, respectively), indicating that the skyrmions have a + *M_z_* core, which corresponds to a skyrmion number *Q* = +1. We reproduce these experimental results using micromagnetic simulations (Experimental Section and Figure S2 for details, Supporting Information), and an antiskyrmion spin texture is observed during the fragmentation of stripe domains; a snapshot of this is captured and presented in Figure 2b(iv). The antiskyrmion, located at the center of the stripe domain (Figure 2b(v)), is then annihilated by thermal fluctuations, leading to the transformation of a stripe domain into two Néel‐type skyrmions, as shown in Figure 2b(vi). For comparison, we also follow the standard field‐sweeping process and simultaneously record the MOKE image, the data is shown in Figure S3 (Supporting Information).

We next analyze the physical principles underlying these observations. Skyrmions and antiskyrmions are stabilized by different forms of DMI. In Heavy Metal/Ferromagnet (HM/FM) polycrystalline or amorphous heterostructures, inversion symmetry is broken only along the direction perpendicular to the interface, resulting in a point group symmetry of *C_nv_*. This symmetry leads to an isotropic DMI, with the energy density expressed as:

(1) $$w_{C_{nv}}=-D_{C_{nv}}\left[\left(M_{z}\frac{\partial M_{x}}{\partial x}-M_{x}\frac{\partial M_{z}}{\partial x}\right)+\left(M_{z}\frac{\partial M_{y}}{\partial y}-M_{y}\frac{\partial M_{z}}{\partial y}\right)\right]$$

where $D_{C_{nv}}$ is the DMI constant for the *C_nv_* system, and *M*_*x*,*y*,*z*_ are the components of the magnetization unit vector in Cartesian coordinates. This equation can be rewritten as:

(2) $$w_{C_{nv}}=-D_{C_{nv}}\left[M_{z}\left(\nabla \cdot M\right)-\left(M\cdot \nabla \right)M_{z}\right]$$

where the divergence gives rise to the spin texture of a Néel‐type skyrmion. The isotropic DMI vector configuration and the resulting Néel‐type skyrmion are illustrated in Figure 2c(i). The DMI vectors *D*_1_, *D*_2_, *D*_3_, *D*_4_ (depicted as blue arrows between neighboring spins *S_i_*,*S_j_* represented by red balls) form a curl and result in the same chirality across all in‐plane directions (isotropic), as evidenced by the spin textures within the two black dashed boxes. This configuration stabilizes a Néel‐type skyrmion. In bulk crystalline non‐centrosymmetric systems, inversion symmetry can be broken along certain crystalline directions, leading to diverse forms of DMI. For example, the inverse tetragonal Heusler compound^[^ ^24^ ^]^ exhibits a *D*_2*d*_ point group symmetry. This symmetry induces an anisotropic DMI, with the energy density expressed as:

(3) $$w_{D_{2d}}=-D_{D_{2d}}\left[\left(M_{z}\frac{\partial M_{y}}{\partial x}-M_{y}\frac{\partial M_{z}}{\partial x}\right)+\left(M_{z}\frac{\partial M_{x}}{\partial y}-M_{x}\frac{\partial M_{z}}{\partial y}\right)\right]$$

where $D_{D_{2d}}$ is the DMI constant for the *D*_2*d*_ system. The anisotropic DMI vector configuration and resulting antiskyrmion are depicted in Figure 2c(ii), with vectors *D*_1_, *D*_3_ pointing inward toward the center atom, and *D*_2_, *D*_4_ pointing outward, thereby producing an opposite chirality. This configuration is visible in the spin textures within the two black dashed boxes and is crucial for stabilizing an antiskyrmion.

However, in contrast to this conventional understanding, we propose a transient antiskyrmion model in our Ta/CoFeB/Ir/MgO heterostructure with a *C_nv_* symmetry group that exhibits isotropic DMI. The experimental evidence that validates this symmetry is presented in Figure S4 and S5 (Supporting Information). To gain further insights into the coexistence of skyrmion and antiskyrmion, we analyze the topology of our system. Two objects are topologically equivalent if they can be continuously deformed into each other. In our study, a single stripe domain is topologically equivalent to a single skyrmion; essentially, the stripe can be seen as a stretched skyrmion. This relationship is illustrated in Phase I of Figure 2d. Both configurations can be mapped once to the unit sphere with a topological number *Q* = −1 (for the case here with − *M_z_* core). In ordered media, such as magnetic mediums, the topological number is usually conserved due to a finite energy barrier, a concept known as topological protection. This phenomenon can be seen in a 2D Heisenberg model with a simplified energy landscape, where only the exchange interaction is considered. As a result, the system's energy minimum is quantized in skyrmion number, which leads to the conservation of the topological number. For a detailed derivation, refer to Supporting Information. Following the above discussion, we assume that the topological number is conserved during the initial transition process. In Figure 2d, Phase I illustrates the initial stripe domain state with *Q* = −1 (bottom picture), which can be mapped onto a unit sphere (middle picture). We thus use a 2D Néel‐type skyrmion cartoon (top picture) to represent its spin texture. Phase II depicts the skyrmion‐antiskyrmion‐skyrmion intertwined state (bottom picture), corresponding to the spin texture in Figure 2b(v). Here, two skyrmions, each with *Q* = −1, form at the two ends of the stripe, adding up to *Q* = −2. To conserve the topological number, a *Q* = +1 antiskyrmion must be created at the center, thereby reducing the total topological number back to the initial state: *Q* = −2 + 1 = −1. The determination of the topological numbers of skyrmions and antiskyrmions is detailed in Experimental Section and Figure S6 (Supporting Information). The mappings of the two end skyrmions and the central antiskyrmion onto unit spheres are shown in the middle picture, while the corresponding spin textures for this phase are depicted in the top cartoon. We now discuss the transition from Phase II to Phase III. As previously mentioned, antiskyrmions require an anisotropic DMI (*D*_2*d*_ or *S*_4_ symmetry) and are thus energetically unfavored in our heterostructure with isotropic DMI (*C_nv_* symmetry). Consequently, the emergence of an antiskyrmion in Phase II increases the system's energy compared to Phase I. This high‐energy state is unstable and is eventually overcome by thermal fluctuations, leading to the annihilation of the central antiskyrmion. As a result, Phase II transitions to Phase III, where two skyrmions are left stable (*Q* = −2), corresponding to the spin texture in Figure 2b(vi). The process transitioning from Phase I to Phase III occurs across all stripe domains, resulting in an increase in skyrmion number, consistent with the observation in Figure 2b(i–iii). We note that the increase in topological number during the stripe domains to two skyrmions transition is consistent with the convention that the net topological charge within an enclosed region is additive.^[^ ^3^ ^]^ We refer to the short presence of the antiskyrmion as the transient antiskyrmion state. In this framework (Figure 2d), we identify the single‐stripe domain state (Phase I) and the two‐skyrmion state (Phase III) as residing within two potential wells, separated by a finite energy barrier *E_B_* created by the transient antiskyrmion (Phase II). The colored arrow indicates the direction of this thermally induced phase transition. The relative energy levels of these phases (*E*_*Phase* *II*_ > *E*_*Phase* *I*_ > *E*_*Phase* *III*_) will be verified in subsequent sections.

### 2.2 Experimental and Theoretical Investigation of Stripe‐to‐Skyrmion Transitions

**Figure 3:**
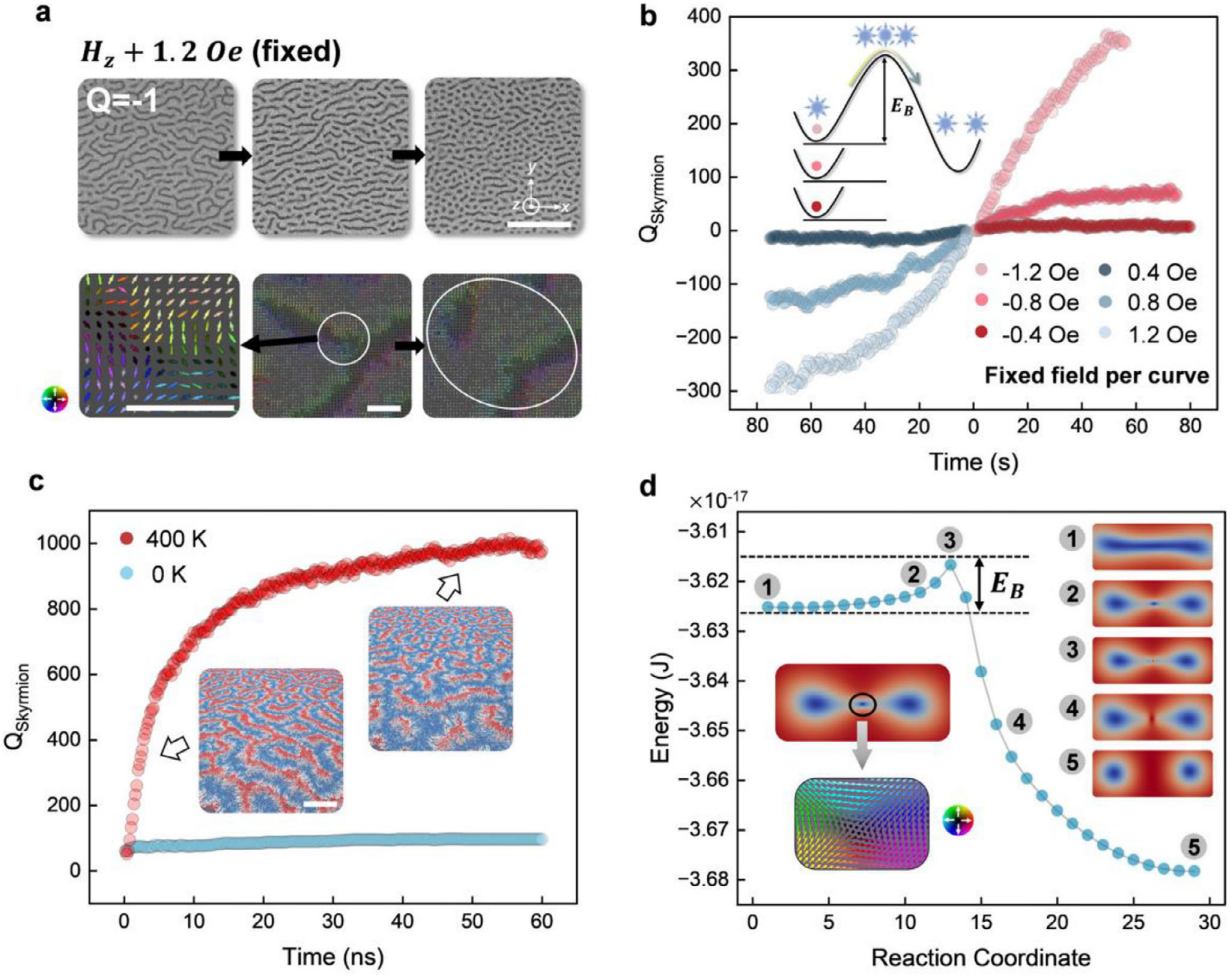
Experimental and theoretical investigation of stripe‐to‐skyrmion transitions. a) MOKE images at + 1.2 *Oe* fixed field showing the evolution of *Q* = −1 stripe domains into skyrmions, alongside simulated spin textures of the associated transient antiskyrmion. Top scale bar: 20 µ*m*; bottom scale bar: 20 *nm*. b) Time evolution of skyrmion numbers extracted from MOKE imaging under different fixed fields. Red (blue) dots represent *Q* = +1(− 1) skyrmions. Inset: Colored dots indicate representative states at − 1.2 *Oe*, −0.8 *Oe*, and − 0.4 *Oe*. As the field approaches zero, the magnetic state resides in a deeper potential well, resulting in a higher energy barrier (*E_B_*). For − 0.8 *Oe* and − 0.4 *Oe*, only the potential wells are shown for visual clarity. Arrow and spin texture symbols follow those used in Figure 2d. c) Time evolution of skyrmion numbers from micromagnetic simulations performed at 400 *K* (red curve) and 0 *K* (blue curve). Insets display representative domain states, with blue indicating − *M_z_*, red + *M_z_*, and white in‐plane spin components. Scale bar: 50 *nm*. d), Calculated minimum energy path and corresponding magnetic states. Blue circles indicate the sequence of magnetic states (reaction coordinates) along the minimum energy path (gray solid line). State ①: Initial single stripe domain; ②: Intermediated skyrmion‐antiskyrmion‐skyrmion configuration; ③ and ④: Transitional states with two stretched irregular shaped skyrmions; ⑤: Final state with two circular skyrmions. Their positions along the energy profile are marked accordingly. *E_B_* denotes the energy barrier separating the initial and final states. Lower inset: Spin texture of the central antiskyrmion in State ②.

The fragmentation phenomena were also observed under varying magnetic field strengths. For example, when the field was held constant at + 1.2 *Oe*, the *Q* = −1 stripe domains, now exhibiting − *M_z_* cores, transformed into discrete skyrmions, as evidenced in **Figure** **3**a. However, at 0 *Oe*, no fragmentation events were observed, and the stripe domains remained stable over a 4h MOKE recording period (Figure S7, Supporting Information). This long‐term stability contrasts sharply with the fragmentation events at ± 1.2 *Oe*, which occur within seconds. According to the proposed topological protection and thermal activation model (Figure 2d), the absence of fragmentation at 0 *Oe* could be attributed to a higher energy barrier that prevents the transition from the single‐stripe state to the two‐skyrmion state. At *H_z_* = 0 *Oe* (Figure 2a), the AHE voltage is positioned at the center of the loop, indicating *M_z_* = 0, which minimizes the demagnetization energy. This stabilization causes the stripe domains to settle into a deeper energy well, effectively increasing the barrier height.

To verify this assumption, we conducted a systematic study at various fixed fields to examine their energy profiles. We quantified the fragmentation events by tracking the time evolution of skyrmion numbers. For tracking technique, see Experimental Section and Figure S8 (Supporting Information) for details. The result is shown in Figure 3b, when the field is maintained at − 1.2 *Oe*, within 60 s, the stripe domains fragmented into over 300 *Q* = +1 skyrmions, eventually forming a skyrmion lattice (Figure S9, Supporting Information). Because each stripe‐to‐skyrmion conversion increases the local topological charge by +1, and many such conversions occur across the film, these local increments accumulate into a global increase in the net topological charge. As the field approached zero, the fragmentation rate decreased. For example, when the field is maintained at − 0.8 *Oe*, the skyrmion number was reduced to ≈100, with the equilibrium state exhibiting a mix of skyrmions and stripes (Figure S9, Supporting Information). At − 0.4 *Oe*, the skyrmion number remained relatively unchanged, with the equilibrium state predominantly featuring stripe domains (Figure S9, Supporting Information). Similar phenomena were observed at positive fields (+ 1.2,   + 0.8,   + 0.4 *Oe* in Figure 3b), but with a reversed skyrmion topology (*Q* = −1). These observations confirm the correlation between fragmentation events and the energy barrier. As the magnetic states approach *M_z_* = 0 at 0 *Oe*, they settle into deeper potential wells by minimizing the demagnetization energy, thereby increasing the energy barrier height. This model is also illustrated in the inset of Figure 3b.

To further validate the topological protection and thermal activation model, we conducted temperature‐dependent micromagnetic simulations (see Experimental Section for details). The magnetic state and the skyrmion number evolution are shown in Figure 3c. The system was initialized with a mixture of stripe domains and skyrmions (left inset image of Figure 3c; Figure S2a, Supporting Information), replicating the experimental conditions. To thermally activate the fragmentation process, finite‐temperature simulations were performed using a fluctuating thermal field. By setting the temperature to 400 *K* (red curve in Figure 3c), the simulation confirmed that stripe domains evolved into skyrmions and eventually formed a quasi‐lattice state (right inset image of Figure 3c; Figure S2a, Supporting Information). These behaviors closely resembled those observed experimentally. Conversely, at 0 *K*, the stripe domains remained stable, with no fragmentation events observed, as evidenced by the blue curve in Figure 3c (or Figure S2b, Supporting Information). This controlled experiment provides strong support for the topological protection and thermal activation model. To quantitatively validate the findings in Figure 3b,c, we performed a simulation at various DMI and field values, as shown in Figure S10 and S11 (Supporting Information).

We further verify the mechanism proposed in Figure 2d by performing a minimum energy path calculation using the geodesic nudged elastic band (GNEB) method (see Experimental Section for detailed parameters). The GNEB method computes the minimum energy path between two fixed equilibrium magnetic states,^[^ ^31^ ^]^ using an atomistic Heisenberg‐type Hamiltonian to determine the energy of these states (reaction coordinates), with reference to the initial state.^[^ ^32^, ^33^ ^]^ The results, shown in Figure 3d, reveal the calculated minimum energy path and corresponding magnetic states for our system (More states on the minimum energy path are presented in Figure S12, Supporting Information). To reproduce the experiment, the initial magnetic state was set as a single stripe domain (State ① in Figure 3d), transitioning to a final state of two skyrmions (State ⑤). The GNEB method determined the energy profile (e.g., ①, ②, ③, ④, and ⑤), confirming that the two‐skyrmion state is energetically favored over the single stripe domain state, thereby enabling the transition from stripe to skyrmion states. An energy barrier (*E_B_*) exists between these two states, requiring an excitation—such as thermal fluctuation in our experiment—to transition from State ① to State ⑤. During this transition, a skyrmion‐antiskyrmion‐skyrmion intertwined state (State ②) emerges, with the central antiskyrmion spin texture shown in the inset. In State ③, the antiskyrmion is annihilated, leaving two stretched, irregularly shaped skyrmions. By State ④, these skyrmions deform toward a more circular shape, achieving more energetic stability. Finally, the two skyrmions reach the equilibrium circular configuration of State ⑤. The minimum energy path calculation results align with our experimental observations and support the mechanism proposed in Figure 2d. A more detailed analysis of the formation mechanism of the antiskyrmion is discussed in Figures S13 and S14 (Supporting Information). Moreover, to further investigate the relationship between energy barrier and antiskyrmion lifetime, we perform calculation and simulation with various PMA values. The results are discussed in Figures S15 and S16 (Supporting Information).

### 2.3 Stripe‐to‐Skyrmion Transitions in a Confined Hall Device

**Figure 4:**
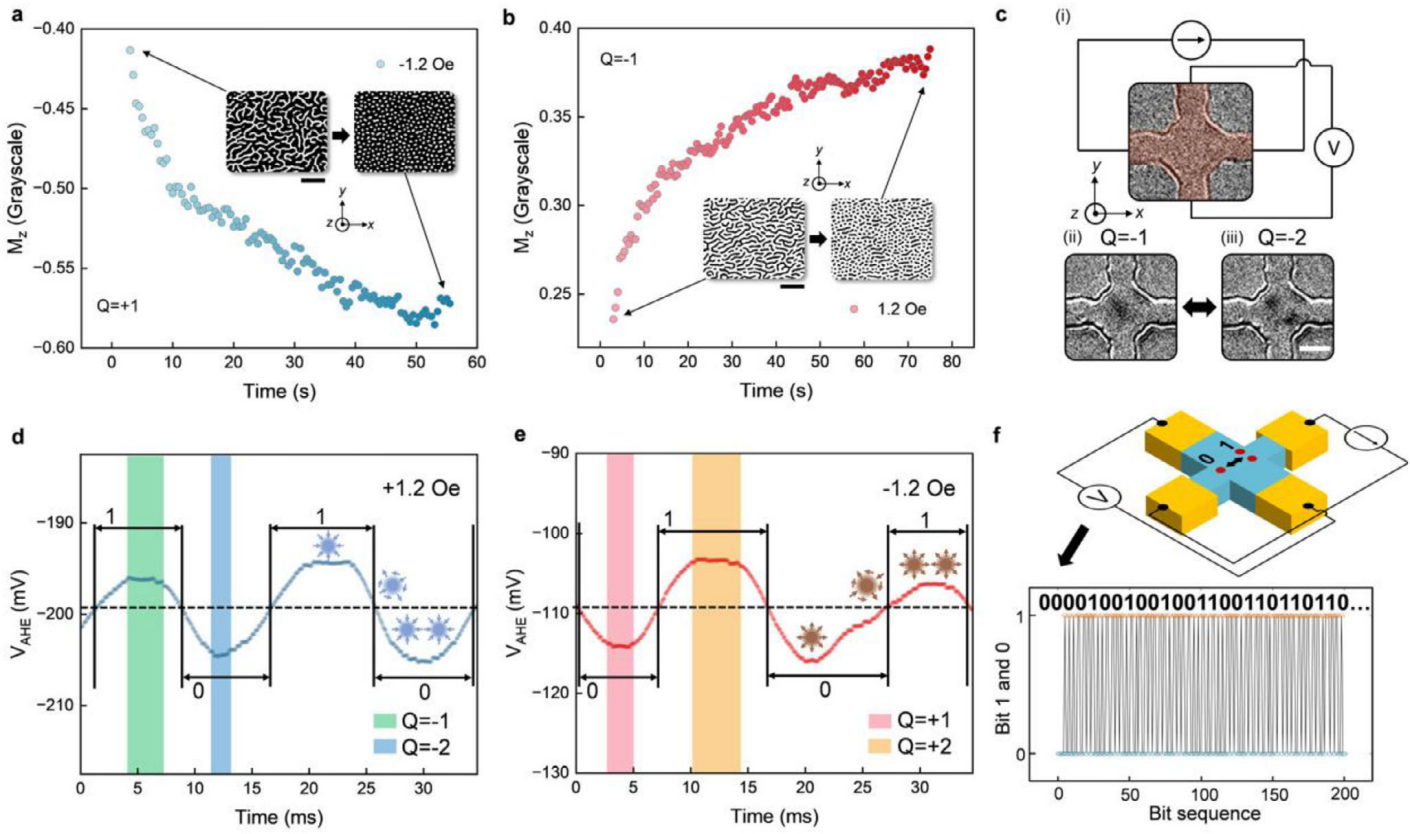
Magnetization dynamics of stripe‐to‐skyrmion transition. a) Time evolution of the *M_z_* or mean grayscale (normalized from [0,  255] to [− 1,   + 1]) for the *Q* = +1 fragmentation at − 1.2 *Oe*. Insets: MOKE images of the initial stripe domain and the final skyrmion lattice. Scale bar: 20 µm. b) Time evolution for the *Q* = −1 case at + 1.2 *Oe*, showing an increase in grayscale (*M_z_*). Insets show MOKE images of the corresponding domain evolution. Scale bar: 20 µm. c) AHE‐based confinement measurements. i) Optical image of the confinement device, with the red region indicating the active area; surrounding areas are substrate. ii) MOKE image showing a single confined stripe domain (black stripe). iii) Transition to a two‐skyrmion configuration (two black circular objects). The transition between ii) and iii) is bidirectional. Note: Edges of the stripe and skyrmions appear blurred due to the limited frame rate of the MOKE imaging. Scale bar: 3 µm. d), AHE measurements at + 1.2 *Oe*. The upper plateaus correspond to the *Q* = −1 single‐stripe state (green‐shaded region), the lower plateaus to the *Q* = −2 two‐skyrmion state (blue‐shaded region), and the transition regions in between represent the transient antiskyrmion state. Each state is illustrated with corresponding spin texture schematics. e) AHE measurements at − 1.2 *Oe* for the *Q* = +1 case. The single‐stripe state locates at the lower plateaus (pink), the *Q* = +2 two‐skyrmion states at the upper plateaus (orange), and the transition regions reflect the transient antiskyrmion. Dashed lines in d,e) represent the average *V_AHE_*, with values above and below defined as bits “1” and “0,” respectively. A 0.1 *ms* sampling interval is used here to resolve temporal dynamics; this rate is not used for random bit collection. f), Top: Schematic of the random number generator. A DC current is applied, and the AHE voltage oscillates between two discrete levels as the system transitions between the single‐stripe state (one red circle) and the two‐skyrmion state (two red circles). Bottom: Raw voltage data sampled at 5 *ms* intervals is binarized into a bitstream by thresholding against the average *V_AHE_* ​. A total of 1.6 × 10^9^ bits were generated; 200 representative bits are shown.

We take a closer look at the evolution of spin texture during the stripe‐to‐skyrmion transition process, specifically focusing on the *Q* = +1 case (+ *z* core spin). During the fragmentation simulation, captured from Figure 2b(iv–vi), the + *z* core spin transient antiskyrmion is annihilated by thermal fluctuations, evolving into the − *z* ferromagnetic background. As a result, the *M_z_* of the system should progressively become more negative as the transition proceeds (through the annihilation of antiskyrmions) and finally reach saturation when fragmentation ceases, and a skyrmion lattice is formed. Conversely, in the *Q* = −1 case (− *z* core spin), during fragmentation and antiskyrmion annihilation, *M_z_* should evolve toward a more positive state. This model, characterized by changes in *M_z_*, was confirmed experimentally. In the experiments, we tracked the evolution of *M_z_* by monitoring changes in the mean grayscale value of the MOKE images. As detailed in the Experimental Section, MOKE images are binarized into white (grayscale value of 255) and black (grayscale value of 0). The mean grayscale value, calculated by averaging the grayscale values of all pixels, quantifies the percentage of the white region in a MOKE image; a value of 255 (0) indicates all pixels are white (black). The *Q* = +1 (*Q* = −1) stripes and skyrmions correspond to regions with a grayscale of 255 (0), respectively. Based on our model, during the *Q* = +1 (*Q* = −1) stripe‐to‐skyrmion transition, the + *z* (− *z*) core spin antiskyrmions annihilate, leading to a decrease (increase) in *M_z_*. This change is reflected by a corresponding decrease (increase) in the mean grayscale of these binarized MOKE images. The evolution of the grayscale values in the MOKE images, captured at magnetic fields of − 1.2 *Oe* and + 1.2 *Oe*, is shown in **Figure** **4**a,b, respectively. For the *Q* = +1 (*Q* = −1) cases, the grayscale indeed decreases (increases), corroborating our observations. This behavior is also reproduced by simulations, as shown in Figure S17 (Supporting Information) and discussed in the Experimental Section.

The experimental observations are consistent with the transient antiskyrmion model; therefore, we correlate changes in *M_z_* with the annihilation of antiskyrmions. To further investigate the dynamics of transient antiskyrmions, we monitor the AHE voltage, *V_AHE_*, which is proportional to the net out‐of‐plane magnetization (*M_z_*). However, there are two notable drawbacks in systems with large dimensions. First, the net *M_z_* change associated with the annihilation of a single antiskyrmion is minimal, as a single antiskyrmion occupies only a small portion of the total space. Second, the simultaneous annihilation of multiple antiskyrmions complicates the extraction of signals associated with a single event. These constraints can be lifted by geometrically confining a single stripe domain, which occupies a substantial portion of the confinement area, ensuring that only single antiskyrmion annihilation occurs. We then conducted experiments and fabricated a confinement device, as shown in Figure 4c(i). The device essentially functions as a Hall crossbar, but with the middle region (red‐shaded area) shrunk down to confine a single stripe domain. Following the same experimental procedures used in the skyrmion fragmentation measurements (Figure 2a), we set the magnetic field from + Saturation to + 1.2 *Oe*. Due to the confinement, a single stripe domain (Figure 4c(ii)) was formed and subsequently transformed into two skyrmions, as seen in Figure 4c(iii). More investigations regarding confinement dimension are presented in Figure S18 (Supporting Information). Micromagnetic simulations of a geometrically confined single stripe show similar behaviors (Figure S19, Supporting Information).

AHE voltage *V_AHE_* is measured to monitor the stripe‐to‐skyrmion transition (see Experimental Section and Figure S20, Supporting Information for details). Surprisingly, upon applying an AHE reading current, we observed that the two skyrmions recombined into a single stripe domain. We suspect this reversion is due to the temperature increase induced by Joule heating. The transition energy barrier from the two‐skyrmion state to the single‐stripe is overcome by increased thermal fluctuations, leading to both forward and reverse topological transitions. The magnetic state oscillates between the single‐stripe and two‐skyrmion states. Based on our model, fragmentation corresponds to the annihilation of an antiskyrmion, whereas recombination, the reverse process, corresponds to its creation. The corresponding *V_AHE_* signal, oscillating between two plateaus, is shown in Figure 4d. The upper plateau, indicated by a green‐shaded rectangle, corresponds to the single‐stripe domain state (*Q* = −1), while the lower plateau, marked by a blue‐shaded rectangle, corresponds to the two‐skyrmion state (*Q* = −2). The transition region between these plateaus corresponds to the transient antiskyrmion state. This identification relies on two criteria: 1. The proportionality of *V_AHE_* to *M_z_*, where *V_AHE_* = −295.8 *mV* at + *M_z_* saturation and 11 *mV* at − *M_z_* saturation, as shown by the min and max values in Figure S20 (Supporting Information). 2. Changes in *M_z_*: During the *Q* = −1 fragmentation (or *Q* = −2 recombination), the annihilation (or creation) of a − *z* core antiskyrmion leads to an increase (or decreases) in *M_z_*, correspondingly decreasing (or increasing) *V_AHE_*. The case for *Q* = +1 (and *Q* = +2) is shown in Figure 4e, with the plateaus and Δ*V_AHE_* reversed. We note that the oscillation amplitude is ≈10 *mV*, while the signal variation in the saturated states (the two plateaus in Figure S20, Supporting Information), without oscillation, is ≈0.27 *mV* (attributed to instrumental noise). This comparison effectively rules out the possibility that the observed signals are due to instrumental noise. We note that, to resolve the dynamics, the analog output of the lock‐in amplifier was used to read out the AHE signal, which amplifies the signal by >100× compared to the raw AHE (Figure 2a).

Upon observing the *V_AHE_* oscillations, we utilize this mechanism to generate stochastic bit streams. The oscillation signal is divided into three parts: the single‐stripe‐domain (|*Q*| = 1) state; the two‐skyrmion‐state (|*Q*| = 2); and the transition region, corresponding to the transient antiskyrmion state. Because of their thermally activated nature, the duration of these states is random, serving as the basis for stochasticity. To generate random numbers, we define the bits “1” and “0”. A feasible approach involves computing the average *V_AHE_* signal of the three states, as indicated by the center dashed line in Figure 4d,e. A bit is defined as “1” if it is above this line and “0” if below. By systematically collecting this bit stream and considering the stochastic nature of this mechanism, this device functions effectively as a random number generator.

To collect the stochastic bit stream of the device (Figure 4c,f top panel), we set the sampling time to half the average duration of the “1” and “0” cycle (5 *ms*), with the raw data presented in Figure S21 (Supporting Information). This data is then compared with the average *V_AHE_* to binarize the bit stream. Currently, this comparison is conducted in silico. For practical applications, it can be implemented using a comparator circuit. A total of 1.6 × 10^9^ bits were collected, and an example sequence of two hundred bits is displayed in Figure 4f lower panel. To verify the randomness of the bit stream, we performed the NIST true random number generator standard test,^[^ ^34^ ^]^ which our data successfully passed (see Supporting Information for details). Additional investigations into the effects of device confinement geometry and shape anisotropy are shown in Figure S22 and S23 (Supporting Information). Additional investigations into tuning the ratio of “1” to “0”, namely the probability of the random number, are provided in Figure S24 (Supporting Information).

### 2.4 Probabilistic Ising Computing

Having established the stochasticity and validated the randomness of bitstreams, we now turn to their use in functional computation. Harnessing physical randomness for computation remains a central challenge in the development of probabilistic hardware systems. In this context, the thermally activated transitions of confined magnetic solitons provide a naturally stochastic resource for sampling‐based approaches.

**Figure 5:**
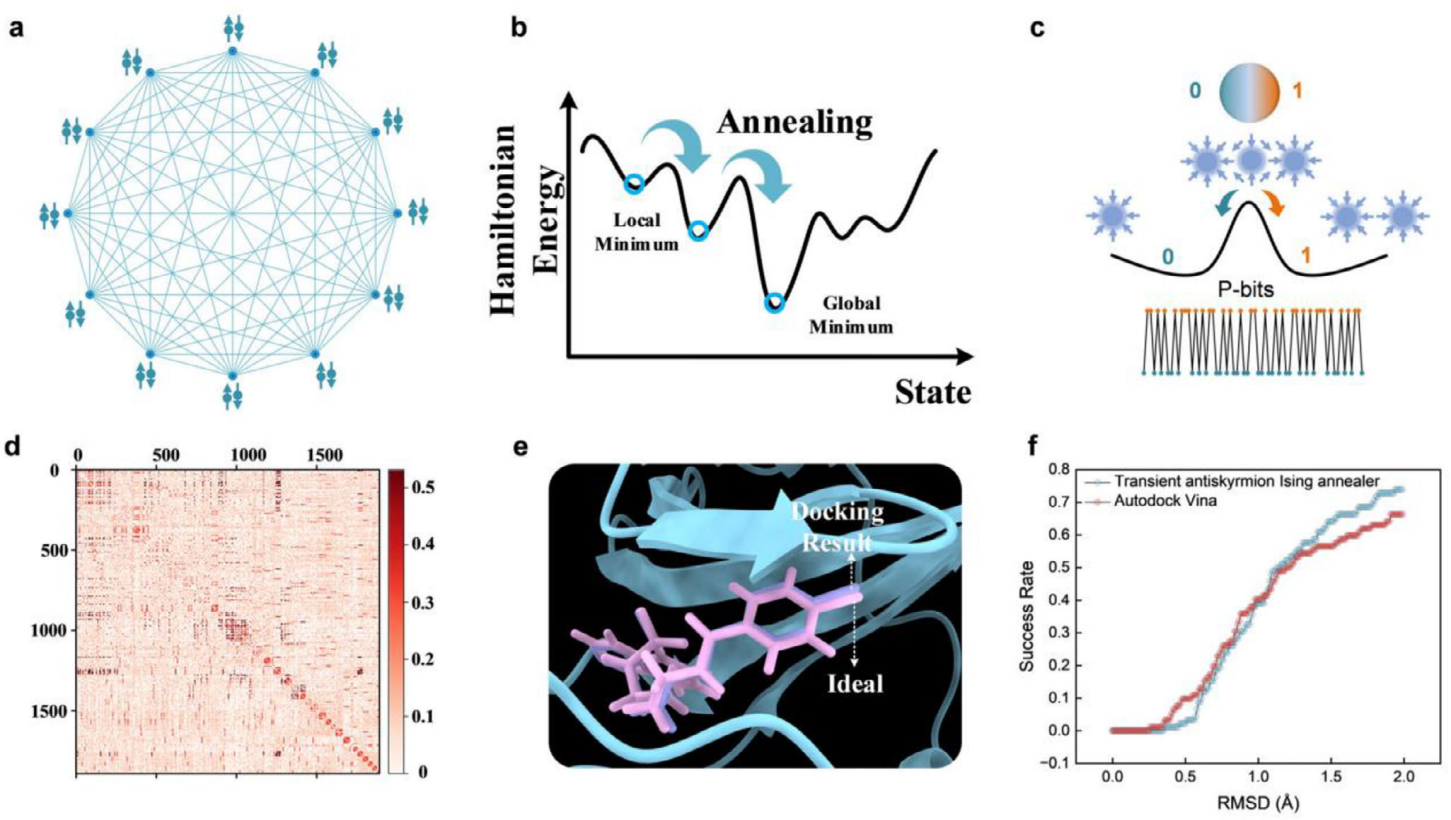
Probabilistic Ising computing using stochastic bits. a) Schematic of a twelve‐spin Ising model with all‐to‐all connectivity. Spin states are indicated by up and down arrows, and interactions between spins are represented by connecting lines. b) SA process used to find the global energy minimum of the Ising system. c) Transient antiskyrmion‐based stochastic bits, utilized as probabilistic inputs in the SA process. d) Weight matrices of the 1892‐bit Ising model used in the molecular docking task for the 1YDT protein. e) Molecular docking result for the 1YDT protein. The pink molecule labeled “Ideal” represents the actual structure observed under an electron microscope, while the purple “Docking Result” shows the output from our Ising SA approach using antiskyrmion‐based bits. f) Docking performance evaluated by the RMSD between predicted binding poses and real crystal structures from the CASF‐2016 dataset, using our transient antiskyrmion‐based Ising solver and the AutoDock Vina software. Here, the success rate indicates the proportion of atoms whose RMSD falls below a given threshold—for example, a 70% success rate means that 70% of the atoms have an RMSD less than 2 Å.

To demonstrate the effectiveness of this physics‐derived randomness, we implemented a probabilistic Ising machine .^[^ ^35^ ^]^ The system is based on an all‐to‐all connected Ising model, where each node represents a binary spin state (up or down) and edges represent pairwise interactions (**Figure** **5**a). Optimization problems are encoded into this network, with solutions corresponding to the model's ground state. A simulated annealing (SA) process is employed to navigate the energy landscape and avoid local minima (Figure 5b), during which the stochastic bitstreams derived from transient antiskyrmion dynamics (Figure 5c) are used as the random sampling input.

To illustrate the computational potential of physics‐derived stochastic bits, we applied our system to a representative optimization task: molecular docking. This problem involves identifying the most energetically favorable binding configuration between a molecule and a protein target. By mapping the docking problem onto an Ising model, we use our stochastic bits as a random input source within a SA framework. Implementation details and derivations are provided in the Supporting Information.

The resulting Ising model is encoded in the form of a weight matrix, with quadratic and linear terms derived from the docking energy landscape. An example of this interaction matrix, used to guide spin updates during optimization, is shown in Figure 5d. Using this solver, we computed the optimal binding pose for the 1YDT molecule, shown in Figure 5e. The predicted result (purple) closely aligns with the experimentally verified structure (pink), indicating high docking accuracy. To benchmark performance, we further evaluated docking outcomes using the CASF‐2016 dataset, comparing results with those obtained from AutoDock Vina—a widely used molecular docking software. As shown in Figure 5f, over 70% of our predicted atom positions achieved a root‐mean‐square deviation (RMSD) below 2 Å, demonstrating accuracy comparable to that of established state‐of‐the‐art methods. These results underscore the viability of our stochastic bitstreams as a functional computational primitive for real‐world optimization problems.

## 3 Conclusion

In conclusion, we have observed the stripe‐to‐skyrmion transition and proposed a skyrmion–antiskyrmion–skyrmion intertwined configuration. We interpret this transition as a consequence of the interplay between topological protection and thermal activation. Through micromagnetic simulations and minimum‐energy‐path calculations, we clarify the creation and annihilation mechanisms of transient antiskyrmions. Importantly, the oscillatory nature of this transition enables the generation of stochastic bitstreams, which we employ in a proof‐of‐concept probabilistic Ising machine for molecular docking.

Our findings suggest several promising directions. The transient antiskyrmion model may extend to other solitonic materials, potentially including anti‐hopfions,^[^ ^7^ ^]^ anti‐merons,^[^ ^36^ ^]^ or higher‐order antiskyrmions.^[^ ^37^ ^]^ In addition, by tuning material parameters such as PMA and DMI, the stability of these spin textures could be adjusted to generate stochastic bitstreams with tailored probability distributions. Such control would support applications in Monte Carlo sampling, Bayesian inference, and stochastic neural networks,^[^ ^38^ ^]^ where nonuniform randomness provides a distinct advantage.

Taken together, our results contribute to the understanding of soliton interactions in magnetic materials and introduce a pathway for linking topological physics with probabilistic functionality. This connection strengthens the foundation for future studies in magnetism and materials engineering, while suggesting opportunities for unconventional spintronic computing.

## Conflict of Interest

The authors declare no conflict of interest.

## Author Contributions

B.D., T.W., A.L., and S.X. contributed equally to this work. B.D. and T.W., and A.L. designed, planned, and initiated studies. B.D. and T.W., and S.X. prepared material samples. B.D. and T.W. conducted the MOKE and transport measurements and analyzed the data. K.W. fabricated the devices. B.D. and T.W. performed the simulations. Z.B., X.Z., J.Y., and Z.L. realized the algorithm of the Ising solver for protein molecule docking and implemented the application experiment. K.L.W. supervised the project. B.D., T.W., A.L., and K.L.W. drafted the manuscript. All authors discussed the results and commented on the manuscript.

## Supporting information



Supporting Information

## Data Availability

The data that support the findings of this study are available from the corresponding author upon reasonable request.

## References

[1] A. M. Kosevich , B. A. Ivanov , A. S. Kovalev , Phys. Rep. 1990, 194, 117.

[2] A. Ore , J. L. Powell , Phys. Rev. 1949, 75, 1696.

[3] N. D. Mermin , Rev. Mod. Phys. 1979, 51, 591.

[4] D. Raftrey , S. Finizio , R. V. Chopdekar , S. Dhuey , T. Bayaraa , P. Ashby , J. Raabe , T. Santos , S. Griffin , P. Fischer , Sci. Adv. 2024, 10, adp8615. 10.1126/sciadv.adp8615 PMC11446272 39356762

[5] T.‐H. Kim , H. Zhao , B. Xu , B. A. Jensen , A. H. King , M. J. Kramer , C. Nan , L. Ke , L. Zhou , Nano Lett. 2020, 20, 4731. 32202799 10.1021/acs.nanolett.0c00080

[6] L. Peng , R. Takagi , W. Koshibae , K. Shibata , K. Nakajima , T.‐H. Arima , N. Nagaosa , S. Seki , X. Yu , Y. Tokura , Nat. Nanotechnol. 2020, 15, 181. 31959930 10.1038/s41565-019-0616-6

[7] F. Zheng , N. S. Kiselev , L. Yang , V. M. Kuchkin , F. N. Rybakov , S. Blügel , R. E. Dunin‐Borkowski , Nat. Phys. 2022, 18, 863.

[8] F. Rendell‐Bhatti , R. J. Lamb , J. W. van der Jagt , G. W. Paterson , H. J. M. Swagten , D. McGrouther , Nat. Commun. 2020, 11, 3536. 32669654 10.1038/s41467-020-17338-7 PMC7363836

[9] Y. Zhou , E. Iacocca , A. A. Awad , R. K. Dumas , F. C. Zhang , H. B. Braun , J. Åkerman , Nat. Commun. 2015, 6, 8193. 26351104 10.1038/ncomms9193 PMC4579603

[10] N. Gao , S.‐G. Je , M.‐Y. Im , J. W. Choi , M. Yang , Q. Li , T. Y. Wang , S. Lee , H.‐S. Han , K.‐S. Lee , W. Chao , C. Hwang , J. Li , Z. Q. Qiu , Nat. Commun. 2019, 10, 5603. 31811144 10.1038/s41467-019-13642-z PMC6898613

[11] F. Muckel , S. von Malottki , C. Holl , B. Pestka , M. Pratzer , P. F. Bessarab , S. Heinze , M. Morgenstern , Nat. Phys. 2021, 17, 395.

[12] R. Juge , N. Sisodia , J. U. Larrañaga , Q. Zhang , V. T. Pham , K. G. Rana , B. Sarpi , N. Mille , S. Stanescu , R. Belkhou , M.‐A. Mawass , N. Novakovic‐Marinkovic , F. Kronast , M. Weigand , J. Gräfe , S. Wintz , S. Finizio , J. Raabe , L. Aballe , M. Foerster , M. Belmeguenai , L. D. Buda‐Prejbeanu , J. Pelloux‐Prayer , J. M. Shaw , H. T. Nembach , L. Ranno , G. Gaudin , O. Boulle , Nat. Commun. 2022, 13, 4807. 35974009 10.1038/s41467-022-32525-4 PMC9381802

[13] J. Tang , Y. Wu , W. Wang , L. Kong , B. Lv , W. Wei , J. Zang , M. Tian , H. Du , Nat. Nanotechnol. 2021, 16, 1086. 34341518 10.1038/s41565-021-00954-9

[14] T. Yokouchi , S. Sugimoto , B. Rana , S. Seki , N. Ogawa , S. Kasai , Y. Otani , Nat. Nanotechnol. 2020, 15, 361. 32231267 10.1038/s41565-020-0661-1

[15] W. Koshibae , N. Nagaosa , Nat. Commun. 2014, 5, 5148. 25322803 10.1038/ncomms6148

[16] S. Woo , K. M. Song , X. Zhang , M. Ezawa , Y. Zhou , X. Liu , M. Weigand , S. Finizio , J. Raabe , M.‐C. Park , K.‐Y. Lee , J. W. Choi , B.‐C. Min , H. C. Koo , J. Chang , Nat. Electron. 2018, 1, 288.

[17] F. Büttner , I. Lemesh , M. Schneider , B. Pfau , C. M. Günther , P. Hessing , J. Geilhufe , L. Caretta , D. Engel , B. Krüger , J. Viefhaus , S. Eisebitt , G. S. D. Beach , Nat. Nanotechnol. 2017, 12, 1040. 28967891 10.1038/nnano.2017.178

[18] S. Woo , K. Litzius , B. Krüger , M.‐Y. Im , L. Caretta , K. Richter , M. Mann , A. Krone , R. M. Reeve , M. Weigand , P. Agrawal , I. Lemesh , M.‐A. Mawass , P. Fischer , M. Kläui , G. S. D. Beach , Nat. Mater. 2016, 15, 501. 26928640 10.1038/nmat4593

[19] L. Caretta , M. Mann , F. Büttner , K. Ueda , B. Pfau , C. M. Günther , P. Hessing , A. Churikova , C. Klose , M. Schneider , D. Engel , C. Marcus , D. Bono , K. Bagschik , S. Eisebitt , G. S. D. Beach , Nat. Nanotechnol. 2018, 13, 1154. 30224795 10.1038/s41565-018-0255-3

[20] F. Büttner , I. Lemesh , G. S. D. Beach , Sci. Rep. 2018, 8, 4464. 29535320 10.1038/s41598-018-22242-8 PMC5849609

[21] J. C. Criado , S. Schenk , M. Spannowsky , P. D. Hatton , L. A. Turnbull , Sci. Rep. 2022, 12, 19179. 36357466 10.1038/s41598-022-22043-0 PMC9649801

[22] A. Fert , P. M. Levy , Phys. Rev. Lett. 1980, 44, 1538.

[23] U. K. Roessler , A. N. Bogdanov , C. Pfleiderer , Nature 2006, 442, 797. 16915285 10.1038/nature05056

[24] A. K. Nayak , V. Kumar , T. Ma , P. Werner , E. Pippel , R. Sahoo , F. Damay , U. K. Rößler , C. Felser , S. S. P. Parkin , Nature 2017, 548, 5. 10.1038/nature23466 28846999

[25] N. Mohseni , P. L. McMahon , T. Byrnes , Nat. Rev. Phys. 2022, 4, 363.

[26] A. Shapiro , Handbooks in *Operations Research* and *Management Science* , 10, Elsevier, Amsterdam, Netherlands 2003, 353.

[27] J. Gawlikowski , C. R. N. Tassi , M. Ali , J. Lee , M. Humt , J. Feng , A. Kruspe , R. Triebel , P. Jung , R. Roscher , M. Shahzad , W. Yang , R. Bamler , X. X. Zhu , Artif. Intell. Rev. 2023, 56, 1513.

[28] A. Alaghi , J. P. Hayes , ACM Trans. Emb. Comput. Syst. (TECS) 2013, 12, 1.

[29] W. A. Borders , A. Z. Pervaiz , S. Fukami , K. Y. Camsari , H. Ohno , S. Datta , Nature 2019, 573, 390. 31534247 10.1038/s41586-019-1557-9

[30] A. Barman , S. Mondal , S. Sahoo , A. De , J. Appl. Phys. 2020, 128, 170901.

[31] X. Chen , E. Chue , J. F. Kong , H. R. Tan , H. K. Tan , A. Soumyanarayanan , Phys. Rev. Appl. 2022, 17, 044039.

[32] D. Cortés‐Ortuño , W. Wang , M. Beg , R. A. Pepper , M.‐A. Bisotti , R. Carey , M. Vousden , T. Kluyver , O. Hovorka , H. Fangohr , Sci. Rep. 2017, 7, 4060. 28642570 10.1038/s41598-017-03391-8 PMC5481343

[33] P. F. Bessarab , V. M. Uzdin , H. Jónsson , Comput. Phys. Commun. 2015, 196, 335.

[34] L. E. Bassham , A. L. Rukhin , J. Soto , J. R. Nechvatal , M. E. Smid , E. B. Barker , S. D. Leigh , M. Levenson , M. Vangel , D. L. Banks , N. A. Heckert , J. Dray , S. Vo , A Statistical Test Suite for Random and Pseudorandom Number Generators for Cryptographic Applications, National Institute of Standards and Technology (NIST), Gaithersburg, MD, USA, 2010.

[35] A. Lucas , Front. Phys. 2014, 2, 5.

[36] X. Z. Yu , W. Koshibae , Y. Tokunaga , K. Shibata , Y. Taguchi , N. Nagaosa , Y. Tokura , Nature 2018, 564, 95. 30518889 10.1038/s41586-018-0745-3

[37] R. Ozawa , S. Hayami , Y. Motome , Phys. Rev. Lett. 2017, 118, 147205. 28430467 10.1103/PhysRevLett.118.147205

[38] R. M. Neal , Bayesian Learning for Neural Networks, Vol. 118, Springer Science & Business Media, New York, 2012.

