# Supplementary material for: Transient Antiskyrmion‐Mediated Topological Transitions in Isotropic Magnets
Source: Adv Sci (Weinh). 2026 Jan 4;13(11):e13126. doi: 10.1002/advs.202513126 (PMC12931202; doi:10.1002/advs.202513126)
Supplement: Supplementary file 1 — Supporting Information [file ADVS-13-e13126-s001.docx]

**Supplementary Materials for**

**Transient Antiskyrmion-Mediated Topological Transitions in Isotropic Magnets**

**Authors**

Bingqian Dai^1,†,*^, Tianyi Wang^1,†^, Albert Lee^1,†^, Shijie Xu^1,†^, Zhongjian Bian^3^, Xinyue Zhu^2,3^, Puyang Huang^1^, Aadi Chaturvedi^4^, Yaochen Li^1^, Yang Cheng^1^, Qingyuan Shu^1^, Haoran He^1^, Hanshen Huang^1^, Lixuan Tai^1^, Kin Wong^1^, Jinbo Yang^2^, Anjan Soumyanarayanan^4^, Zhaochu Luo^2,*^, and Kang L. Wang^1,*^

**Affiliations**

*^1^Department of Electrical and Computer Engineering, University of California, Los Angeles, California 90095, United States.*

*^2^State Key Laboratory of Artificial Microstructure and Mesoscopic Physics, Institute of Condensed Matter Physics and Materials, School of Physics,* *Peking University, 100871 Beijing, China.*

*^3^ICY Technology (Beijing) Co., Ltd. 100871 Beijing, China.*

*^4^Department of Physics,* *National University of Singapore, Singapore 117551, Singapore.*

^†^These authors contributed equally to this work.

Corresponding author. E-mail: ^*^[bdai@g.ucla.edu](mailto:bdai@g.ucla.edu), ^*^[zhaochu.luo@pku.edu.cn](mailto:zhaochu.luo@pku.edu.cnm), [^*^wang@ee.ucla.edu](mailto:*wang@ee.ucla.edu)

**This Supplementary file includes:**

Experimental Section

Topological number conservation

NIST true random number generator standard test

Molecular docking implementation details

Figs. S1 to S26

Table. S1

**Experimental Section**

**Material growth and device fabrication**

Ta ($5 nm$) / Co_40_Fe_40_B_20_ ($1 nm$) / Ir ($0.03-0.15 nm$) / MgO ($2 nm$) / Al_2_O_3_ ($5 nm$) heterostructures (Supplementary **Fig. S1a**), with a nominal thickness in $nm$, was grown on semi-insulating Si substrate with a $100 nm$ thermal oxide by dc and radio-frequency magnetron sputtering at room temperature in an AJA International Physical Vapour Deposition System. The base pressure was $<1 \times{10}^{-8} torr$ and the deposition Ar pressure was $3 mtorr$. The Ta bottom layer acts as a buffer and SOC source, the CoFeB layer serves as the ferromagnetic medium, and the MgO layer is used to provide the CoFeB/MgO interface to induce the perpendicular magnetic anisotropy. The thin (dusting) Ir layer, approximately one atomic layer thick, is sandwiched between the CoFeB and MgO layers to fine-tune the perpendicular magnetic anisotropy. This Ir layer displays a wedge-like geometry, with its thickness varying continuously from $0.03 nm$ to $0.15 nm$ and a gradient of approximately $0.02 nm$ per $1 cm$ along the sample's length. The structure is capped with a Al_2_O_3_ top layer that serves as a protective barrier against oxidation. The films were then post-annealed in vacuum at $250 ^{\circ}C$ for $30$ minutes to enhance the perpendicular magnetic anisotropy. The material stacks were then patterned into Hallbar and confinement device structures using standard photolithography, dry etching, wet etching, and evaporation technique.

**Tuning perpendicular magnetic anisotropy**

The perpendicular magnetic anisotropy (PMA) of the system is fine-tuned by the Ir insertion thickness. As a result, the domain states evolve from large multiple domains (high PMA) to skyrmion lattice (low PMA). To obtain the domain states along the Ir gradient, the film was initially saturated using a large positive magnetic field ($+H_{z}^{Sat}$). The magnetic field was then swept from this positive saturation to negative saturation ($-H_{z}^{Sat}$) while capturing the MOKE images. The images that best depict the domain states are presented in Supplementary **Fig. S1b**. Five locations along the direction of the Ir thickness gradient were examined. It is observed that as the Ir layer increases in thickness, the domain states transition from large multiple domains (i) to a labyrinth domain (ii), evolving into a denser labyrinth domain (iii & iv), and ultimately forming a skyrmion lattice (v). This evolution indicates a decrease in PMA with increasing thickness of the Ir layer. Our investigation was focused on the location with an Ir thickness of $0.15 nm$.

**Polar magneto-optic Kerr effect measurement**

Polar Magneto-Optic Kerr Effect (MOKE) imaging experiments were conducted using a custom-built wide-field MOKE microscope, offering spatial ($360 nm$ resolution) and temporal ($20 ms$resolution) capabilities. The external magnetic field was produced by a GMW-5201 Helmholtz coil, powered by a Kepco BOP 5-20D supply. After applying a large positive out-of-plane magnetic field $+H_{z}$ to saturate the sample, a background image was acquired. Subsequent images were differentially obtained by subtracting this background image, providing magnetic contrast. Polar MOKE signal is proportional to the net magnetization in the out-of-plane direction ($M_{z}$). The white (black) color indicates a positive (negative) $M_{z}$.

**Micromagnetic simulations of skyrmion fragmentation and transient antiskyrmions**

To reproduce the experimental results, micromagnetic simulations were performed with the MuMax3 code (*39*) using a world size of $400\times400\times1$ cells with cell size of $2\times2\times1 {nm}^{3}$ and the following material parameters: exchange constant ${10}^{-11} J/m$, saturation magnetization ${9\times10}^{5} A/m$, out-of-plane uniaxial anisotropy ${5\times10}^{5} J/m^{3}$, interfacial DMI ${3\times10}^{-3} J/m^{2}$, out-of-plane magnetic field $-0.3 T$. 400 K and 0 K cases are simulated as controlled experiments. The finite temperature effect is achieved by the fluctuating thermal field embedded in MuMax3. We initialize the system with a mixture of stripe domains and skyrmions as shown in Supplementary **Fig. S2a**, similar to the experimental condition. To induce the fragmentation phenomena, we employ the finite temperature simulation using the fluctuating thermal field embedded in MuMax3. The temperature is set at $400 K$ and the fragmentation events indeed occur, from Supplementary **Fig. S2a**, we can see the stripes are fragmented into skyrmions, and finally form a quasi-lattice state. These behaviors resemble the experimental observations. If the temperature is set at $0 K$. The stripe domains remain stable and no fragmentation events happen, as shown in Supplementary **Fig. S2b**. This observation confirms the fragmentation are caused by thermal activation.

**Calculation of topological numbers for skyrmions and antiskyrmions**

The topology of skyrmion can be characterized by skyrmion number $Q=\frac{1}{4\pi}\iint\boldsymbol{M}\cdot(\frac{\partial\boldsymbol{M}}{\partial x}\times\frac{\partial\boldsymbol{M}}{\partial y})dxdy$. $Q$ can be further broken down to the product of skyrmion polarity and vorticity (*40, 41*). The in-plane projection of skyrmion or antiskyrmion spin is depicted by the black arrows in Supplementary **Fig. S6**. Vorticity $(\nu)$ is defined as the winding number of these in-plane spins. For skyrmion, if we count the in-plane spin rotation direction in a clockwise fashion, it winds around the unit circle once clockwise, giving rise to winding number or vorticity $\nu=+1$. While for antiskyrmion, it winds also once but in a counterclockwise way, giving rise to $\nu=-1$. The vorticity is illustrated as the red circle-with-arrow. Polarity $(p)$ is defined by the direction of center spin. If the center spin points in the $+z(-z)$ direction, the polarity $p=+1(-1)$. The polarity is illustrated as the blue circle-with-cross, indicating the center spin is pointing into the paper $(-z)$. For the skyrmion, it has $p=-1,\nu=+1$, giving rise to $Q=p\times\nu=-1$. While for antiskyrmion, it has $p=-1,\nu=-1$, giving rise to $Q=p\times\nu=+1$.

**Techniques for tracking the time evolution of skyrmion numbers**

The tracking is achieved by Fiji (*42*), a distribution of the well-known open-source software ImageJ. The tracking process is depicted in Supplementary **Fig. S8**, with the case at a set field of $-1.6 Oe$ serving as an example. The fragmentation process was captured using MOKE images taken at a frame rate of $0.5$ seconds, beginning one second after the field was set. The MOKE images of the initial frames are shown in Supplementary **Fig. S8a(i)** and marked as time zero. These 8-bit grayscale images were subsequently thresholded and binarized using Fiji's built-in tools, as demonstrated in b(i). In our MOKE images, which measure $465\times465$ pixels, a skyrmion is identified as a quasi-circular object occupying an area between $50$ to $200$ pixels. Skyrmions are therefore identified by counting objects within this pixel range, with the counting performed using Fiji’s built-in programs, and the results displayed in c(i). Only quasi-circular objects within the specified pixel area are identified as skyrmions, with red numbers marking their labels. The tracking of skyrmion numbers from frame No. 68 (at 34 seconds) and frame No. 150 (at 75 seconds) is presented in a(ii)-b(ii)-c(ii) and a(iii)-b(iii)-c(iii), respectively. It is important to note that the white triangles at the four corners of Supplementary **Fig. S8a** and **Fig. S8b** are artifacts created by the field diaphragm of the MOKE microscope and do not impact the skyrmion number tracking results. Additionally, the counting of skyrmions is confined to the observation window measuring $90 \mu m$ by $70 \mu m$.

**Parameters for calculating the minimum energy path**

The topological transition mechanism is verified by performing minimum energy path calculation using the geodesic nudged elastic band method implemented by the Fidimag package (*43*). The calculation is performed using world size of $200\times200\times1$ cells with cell size of $2\times2\times1 {nm}^{3}$ and the following material parameters: exchange constant $1.3\times{10}^{-11} J/m$, saturation magnetization ${8.6\times10}^{5} A/m$, out-of-plane uniaxial anisotropy ${3.25\times10}^{4} J/m^{3}$, interfacial DMI ${1.3\times10}^{-3} J/m^{2}$, out-of-plane magnetic field $0.0754 T$.

**Simulation of** $\boldsymbol{M}_{\boldsymbol{z}}$ **changes due to antiskyrmion annihilation**

The change in $M_{z}$ due to annihilation of antiskyrmion is reproduced by simulation. Despite the smaller world size used to reduce computation time, this simulation employs the same parameters as in the previous section. The smaller world size does not impact the physics of the fragmentation phenomena. Supplementary **Fig. S17a** displays the initial and final states for both (i) $Q=+1$ and (ii) $Q=-1$ cases, where the fragmentation events occur, and the $+z$ and $-z$ core spin antiskyrmions are annihilated in (i) and (ii), respectively. Consequently, $M_{z}$ indeed evolves into a more negative (positive) state for $Q=+1 (Q=-1)$ case, as shown in Supplementary **Fig. S17b**. Because of the topology of (anti)skyrmions, the net in-plane magnetization ($M_{x}$ and $M_{y}$) ideally remains at zero. The small fluctuations around zero in Supplementary **Fig. S17b** arise from thermal agitation.

**Micromagnetic simulation of geometrically confined single antiskyrmion dynamics**

Besides a smaller world size of $50\times50\times1$ cells, we use the same parameters as in previous micromagnetic simulations. Supplementary **Fig. S19** shows the time evolution of $M_{z}$ associated with a single fragmentation event. The $M_{z}$ of the system changes from $-0.63$ to $-0.75$ upon the annihilation of a single antiskyrmion, as indicated by the increase in skyrmion number $Q_{Skyrmion}$ from $1$ to $2$. We also observe that the $Q_{Skyrmion}$ is not precisely quantized at $1$ or $2$, due to the thermally induced spin texture fluctuations.

**AHE measurement details**

The measurements were conducted using a Keithley 6221 current source, coupled with either a Keithley 2182A nano-voltmeter (for Hallbar device) or a Stanford Research Systems SR830 lock-in amplifier (for confinement device). The external magnetic field was produced by a GMW-5201 Helmholtz coil, powered by a Kepco BOP 5-20D supply.

To accurately probe the dynamics of transient antiskyrmions, we first need to calibrate the field ramp-up and stabilization time of the Helmholtz coil. This step is crucial to decouple these artifacts from the actual transient antiskyrmion signals. The field-driven magnetization switching occurs on a timescale from microseconds to nanoseconds (*30*). The dynamics of $M_{z}$, and thus the $V_{AHE}$ signal, immediately follow the field changes. Therefore, we use $V_{AHE}$ to calibrate the field ramp-up and stabilization time. The results are shown in Supplementary **Fig. S20**, where the field is set from $+3 Oe$ to $-3 Oe$ ($\pm$Saturation) while $V_{AHE}$ is recorded. The field ramp-up and stabilization times are obtained from the $V_{AHE}$ transition region between the first plateau (when the field is stable at $+3 Oe$, $M_{z}$ is at $+$saturation and $V_{AHE}=-295.8 mV$ ) and the second plateau (when the field is stable at $-3 Oe$, $M_{z}$ is at $-$saturation and $V_{AHE}=11 mV$). The transition region is marked by a blue-shaded rectangle, with the ramp-up and stabilization time determined to be $18.8 ms$. We note that to increase the signal-to-noise ratio, the Keithley 6221 and SR830 are employed here. $V_{AHE}$ is measured from the SR830’s analog output, which has a bandwidth of $100 kHz$ and updates at $256 kHz$. The $V_{AHE}$ reading is amplified by the SR830 from $2.1 mV$ (as shown in Main Text **Fig. 2a**) to $306.8 mV$ (as shown in Supplementary **Fig. S20**), while the proportionality to $M_{z}$ still holds.

**Statistical Analysis**

Statistical evaluation of the generated stochastic bitstreams was performed using the NIST SP 800-22 randomness test suite. Bitstreams were analyzed without additional preprocessing, and pass/fail criteria followed the standard NIST significance threshold. All statistical analyses were performed using the official NIST software package.

**Topological number conservation**

The topological protection can be seen in a simplified energy landscape where only exchange interaction is considered. The following discussion is mostly based on (*44*). The exchange energy of a 2D Heisenberg model (a 2D sheet of spins with SO(3) symmetry) can be written as:

$$\begin{aligned} E=\int A\left[ {(\frac{\partial\boldsymbol{M}}{\partial x})}^{2}+{(\frac{\partial\boldsymbol{M}}{\partial y})}^{2} \right]dxdy\#\left( 1 \right) \end{aligned}$$

Where $A$ is the exchange constant. We expand the following simple inequality:

$$\begin{aligned} {(\frac{\partial\boldsymbol{M}}{\partial x}\pm M\times\frac{\partial\boldsymbol{M}}{\partial y})}^{2}\geq0 \to{(\frac{\partial\boldsymbol{M}}{\partial x})}^{2}+({\boldsymbol{M}\times\frac{\partial\boldsymbol{M}}{\partial y})}^{2}\geq\mp2(\frac{\partial\boldsymbol{M}}{\partial x})\cdot(\boldsymbol{M}\times\frac{\partial\boldsymbol{M}}{\partial y})\#\left( 2 \right) \end{aligned}$$

Because $\frac{\partial\boldsymbol{M}}{\partial x}(\frac{\partial\boldsymbol{M}}{\partial y})\perp\boldsymbol{M}$, we have $({\boldsymbol{M}\times\frac{\partial\boldsymbol{M}}{\partial y})}^{2}=({\frac{\partial\boldsymbol{M}}{\partial y})}^{2}$. The inequality becomes:

$$\begin{aligned} {(\frac{\partial\boldsymbol{M}}{\partial x})}^{2}+({\frac{\partial\boldsymbol{M}}{\partial y})}^{2}\geq\pm2\boldsymbol{M}\cdot(\frac{\partial\boldsymbol{M}}{\partial x}\times\frac{\partial\boldsymbol{M}}{\partial y})\#\left( 3 \right) \end{aligned}$$

The energy of the system thus has the following relation:

$$\begin{aligned} E=\int A\left[ {(\frac{\partial\boldsymbol{M}}{\partial x})}^{2}+{(\frac{\partial\boldsymbol{M}}{\partial y})}^{2} \right]dxdy\geq\int\pm2\boldsymbol{M}\cdot\left( \frac{\partial\boldsymbol{M}}{\partial x}\times\frac{\partial\boldsymbol{M}}{\partial y} \right)dxdy\#\left( 4 \right) \end{aligned}$$

We have skyrmion number: $Q=\frac{1}{4\pi}\iint\boldsymbol{M}\cdot(\frac{\partial\boldsymbol{M}}{\partial x}\times\frac{\partial\boldsymbol{M}}{\partial y})dxdy$, where $Q$ is an integer.

Inequality $\left( 4 \right)$ can be rewritten as:

$$\begin{aligned} E\geq8\pi A|Q|\#\left( 5 \right) \end{aligned}$$

The energy minimum of the system is thus quantized by the skyrmion number. The energy landscape is illustrated in **Fig. S25**, assuming the system stays in the energy minimum. We can see that there is a finite energy barrier from low to high skyrmion number states, leading to the topological protection and conservation of topological number (not increasing).

**NIST true random number generator standard test**

To verify that the transient antiskyrmion bits are truly random, they must pass fifteen NIST RNG tests (*34*). It is important to note that these NIST tests are specially designed to access the randomness of data sets with a 50%-50% unbiased distribution. However, our transient antiskyrmion bits exhibit intrinsic bias due to the two inequivalent potential wells associated with the single-stripe and two-skyrmions states. To address this, our data are processed through four XOR gates to remove the intrinsic bias. Consequently, the bit count is reduced from the original $1.6\times{10}^{9}$ to $1\times{10}^{8}$. These $1\times{10}^{8}$ bits then undergo the NIST tests, successfully passing all fifteen. The results are detailed in Table. S1 below.

The tests serve various purposes. For example, the Frequency test aims to determine whether the number of ones and zeros in a sequence are approximately the same as would be expected for a truly random sequence. Meanwhile, the Block frequency test assesses whether the frequency of ones in an M-bit block is approximately M/2. Detailed descriptions of each test are available in reference (*34*).

**Molecular docking implementation details**

We have selected molecular (protein) docking (*45*) as our target computation task. The primary objective is to determine how effectively a particular molecule can bind to a protein.

1. The stability of molecular docking can be described by the binding free energy $\Delta G_{Bind}$: the lower the docking free energy, the higher the stability of docking. Suppose the ligand $L$ is composed of atoms $p_{1}, p_{2},\ldots, p_{n}$ with coordinates (three-dimensional vectors) $c_{1}, c_{2},\ldots, c_{n}$, respectively. Molecular docking aims to minimize (objective function):

$$\begin{aligned} \Delta G_{Bind}\left( p_{1}, p_{2},\ldots, p_{n}, c_{1}, c_{2},\ldots, c_{n} \right), c_{i}\in D\#\left( 6 \right) \end{aligned}$$

where $D$ is the docking domain (Docking Box, used to restrict ligand poses).

To map this to an Ising model, we discretize the space and convert atomic positioning into binary decision variables. The corresponding Ising Hamiltonian is:

$$\begin{aligned} H= -\left( \sum_{i<j} J_{ij}s_{i}s_{j}+ \sum h_{i}s_{i} \right) \\ I_{i}= \sum J_{ij}s_{j}+ h_{j} \\ s_{i}=sgn \left[ \tanh\left( \beta I_{i} \right)- {rand}_{U}\left( 0,1 \right) \right]\#\left( 7 \right) \end{aligned}$$

Where $s_{i}$ and $s_{j}$ are the Ising spins, $J_{ij}$ the weight matrix, $h_{i}$ the bias field, $I_{i}$ the negative of the partial derivative of $H$ with respect to $s_{i}$, $\beta$ the reciprocal of the temperature coefficient, and ${rand}_{U}\left( 0,1 \right)$ the random variable uniformly distributed between $0$ and $1$. The quadratic terms ($s_{i}s_{j}$) can be translated to weight matrix ($J_{ij}$), linear terms ($s_{i}$) and bias vector ($h_{i}$). The connections between Ising spins are represented as weight matrices, which are shown in **Fig. 5d** of the main text.

Equation (7) is minimized through the simulated annealing (SA) method, with random numbers serving as the foundation for the algorithm's execution. During each iteration of the SA process, a new state is proposed (e.g., by flipping a spin in the Ising model). The energy change $\Delta E$ associated with the transition from the current state to the new state is calculated. To decide whether to accept or reject this state, our transient antiskyrmions are used. It produces stochastic bits which are then transformed into uniform random numbers $r$ in the range $[0, 1]$. The acceptance probability, $P$, for this state is defined by the equation:

$$\begin{aligned} P=e^{(-\Delta E/k_{B}T)}\#\left( 8 \right) \end{aligned}$$

Where $k_{B}$ is the Boltzmann constant and $T$ is the temperature. If $r\leq P$, the new state is accepted, even if $\Delta E>0$ (i.e., the energy increases). This allows the system to potentially escape from local minima. If $r>P$, the new state is rejected, especially as $T$ decreases and fewer higher-energy states are accepted. This probabilistic acceptance mechanism is essential in finding global or near-global minima in complex optimization landscapes. By leveraging the convergence of random number probabilities, the energy of the physical system is reduced to its minimum. True random numbers facilitate the transition from local optima to the global optimum. The quality of these random numbers—specifically their randomness and generation speed—directly impacts the efficiency of finding the global optimum solution.

2. By discretizing space (dividing space into small blocks, each containing state information to characterize whether atoms are present and their types), molecular docking is transformed into a matching problem between ligand atoms and spatial positions (the objective function of this problem is an approximation of the objective function Equation (6)). Suppose the docking domain $D$ is discretized into grid points $g_{s_{1}}, g_{s_{2}},\ldots, g_{s_{n}}$, and $p_{i}$ matches $g_{s_{i}}$, the distance between $p_{i}$ and $p_{j}$ is $d_{ij}$, and the distance between $g_{s_{i}}$ and $g_{s_{j}}$ is $r_{s_{i}s_{j}}$. The mathematical description is:

$$\begin{aligned} min\Delta G_{Bind}\left( p_{1}, p_{2},\ldots, p_{n}, c_{1}, c_{2},\ldots, c_{n} \right)\approx\\ \Delta G_{Bind}^{'}\left( p_{1}, p_{2},\ldots, p_{n}, g_{s_{1}}, g_{s_{2}},\ldots, g_{s_{n}} \right)=\sum_{i=1}^{n} \beta_{p_{i}g_{s_{i}}} \\ s.t. |d_{ij}-r_{s_{i}s_{j}}|\leq c_{dist} \\ s_{i}\in(1,2,\ldots,m)\#\left( 9 \right) \end{aligned}$$

Algorithms used:

- GPM algorithm: In the docking domain $D$, $m$ grid points are generated, and $n$ atomic state bits are assigned to each grid point to represent whether $p_{i}$ matches grid point $g_{s_{j}}$, $i \in(1, 2, ..., n), j \in(1, 2, ..., m)$.
- FAM algorithm: In the docking domain $D$, $m$ grid points are generated, and 3 atomic state bits are assigned to each grid point to represent whether C atoms, H atoms, and O atoms match grid point $g_{s_{j}}$, $j \in(1, 2, ..., m)$.

3. Using $x_{ij}$ as a binary decision variable to represent whether $p_{i}$ matches $g_{s_{j}}$, thereby describing this matching problem as a constrained QUBO model (*46-48*):

$$\begin{aligned} min\Delta G_{Bind}^{'}\left( p_{1}, p_{2},\ldots, p_{n}, g_{s_{1}}, g_{s_{2}},\ldots, g_{s_{n}} \right)= \\ \Delta G_{Bind}^{''}\left( p_{1}, p_{2},\ldots, p_{n}, g_{s_{1}}, g_{s_{2}},\ldots, g_{s_{n}},x_{11},x_{12,}\ldots,x_{nm} \right)=\sum_{i=1}^{n} \sum_{j=1}^{m} \beta_{p_{i}g_{s_{i}}}x_{ij}^{2} \\ s.t. \left| d_{ik}-r_{s_{j}s_{l}} \right|\leq c_{dist} and \sum_{i=1}^{n} x_{ij}\leq1 \\ x_{ij}=\left\{ \begin{aligned} 1,a_{i} matches g_{s_{j}} \\ 0,a_{i} does not match g_{s_{j}} \end{aligned} \right. \\ i,k\in\left( 1,2,\ldots,n \right) and j,l\in\left( 1,2,\ldots,m \right)\#\left( 10 \right) \end{aligned}$$

4. Eliminating the constraints in the QUBO model by introducing Lagrange terms (*46-48*):

$$\begin{aligned} \begin{aligned} min\Delta G_{Bind}^{''}\left( p_{1}, p_{2},\ldots, p_{n}, g_{s_{1}}, g_{s_{2}},\ldots, g_{s_{n}},x_{11},x_{12,}\ldots,x_{nm} \right)= \\ \sum_{i=1}^{n} \sum_{j=1}^{m} \beta_{p_{i}g_{s_{i}}}x_{ij}^{2}+\lambda_{dist}\sum_{i=1}^{n} \sum_{j=1}^{m} \sum_{k=1}^{n} \sum_{l=j+1}^{m} u_{ijkl}x_{ij}x_{kl}+\lambda_{mono}\sum_{i=1}^{n} \sum_{j=1}^{m} \sum_{k=1}^{n} \sum_{l=j+1}^{m} v_{ijkl}x_{ij}x_{kl} \\ u_{ijkl}=\left\{ \begin{aligned} 1,\left| d_{ik}-r_{s_{j}s_{l}} \right|>c_{dist} \\ 0,\left| d_{ik}-r_{s_{j}s_{l}} \right|\leq c_{dist} \end{aligned} \right. \\ v_{ijkl}=\left\{ \begin{aligned} 1,j=l and i\neq k \\ 0,,otherwise \end{aligned} \right. \\ \#\left( 11 \right) \end{aligned} \end{aligned}$$

These processes are summarized in the block flow diagram, as shown in **Fig. S26**.

**Fig. S1.**

| **** |
| --- |
| **Fig. S1 \| Material system and domain evolution. (a),** Schematic of the Ta($5 nm$)/Co_40_Fe_40_B_20_($1 nm$)/Ir($0.03-0.15 nm$)/MgO($2 nm$)/Al_2_O_3_($5 nm$) heterostructures. **(b),** Domain evolution across a sample with an Ir gradient. The sputtering sample measures $1\times6 {cm}^{2}$. Domain evolution is achieved by fine-tuning the PMA through Ir insertion thickness. Five representative locations are presented. |

**Fig. S2.**

| **** |
| --- |
| **Fig. S2 \| Reproduce the experiment by micromagnetic simulations.** **(a),** The temperature is set at $400 K$, fragmentation events occur. **(b),** At $0 K$, no fragmentation happens. |

**Fig. S3.**

We follow the standard field-sweeping process with each field held for 0.5 second and simultaneously recording the MOKE image, the data is shown in **Fig. S3**. **Fig. S3(a)** presents the full hysteresis loop with numbers labelling the representative states, **Fig. S3(b)** presents the MOKE images of these nine representative states, we can see that following the hysteresis loop, the system evolves from +z direction saturation state (①) to Q=-1 isolated skyrmion states (②, ③), then to Q=-1 stripe-skyrmion mixed state (④), then to pure stripe domain state (⑤), then to Q=+1 stripe-skyrmion mixed state (⑥), then to Q=+1 isolated skyrmion states (⑦, ⑧), finally to -z direction saturation state (⑨). We note that here we do not observe skyrmion lattice state because each field is only held for 0.5 s, in such a short period, only a few stripes are fragmented into skyrmions, thus the skyrmion lattice is not formed.

| **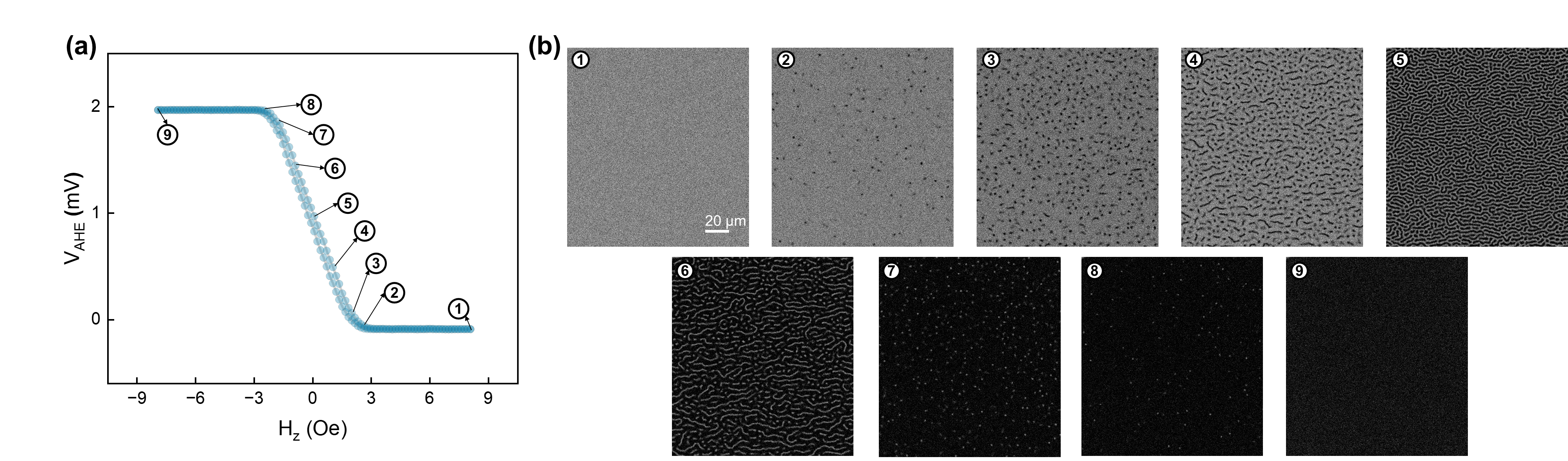** |
| --- |
| **Fig. S3 \| MOKE images recording during the hysteresis loop sweeping. (a),** Hysteresis loop and the label number of the representative magnetic states. **(b),** MOKE images of the corresponding magnetic states. |

**Figs. S4 & S5.**

**Discussion of the symmetry of our system**

As discussed in the Main Text, an antiskyrmion can emerge in an isotropic DMI system with Cnv symmetry, which normally favors skyrmions rather than antiskyrmions. Consequently, the antiskyrmion can only appear as an unstable, transient state. This represents one of our key insights, as it challenges the conventional view that antiskyrmions are stabilized exclusively by anisotropic DMI in D2d or S4​ systems.

It is therefore necessary to verify that our system exhibits Cnv ​symmetry, or more precisely C∞v symmetry, which belongs to the amorphous or weakly polycrystalline films. In such systems, the lack of long-range crystallographic order eliminates any significant in-plane anisotropy arising from the crystal lattice. As a result, the film exhibits effective rotational invariance about the film normal and mirror symmetry across vertical planes—symmetries that are well captured by the C∞v group.

Our material stack is as follows:

Si (0.5mm)/SiO_2_ (100nm)/Ta (5nm)/CoFeB (1nm)/Ir(0.03nm-0.15nm)/MgO(2nm)/Al_2_O_3_(5nm).

The Si substrate has a (100) crystal orientation. The thermally oxidized SiO_2_​ serves as an amorphous buffer layer. Ta is the heavy metal layer, CoFeB is the ferromagnetic layer, MgO is the oxide layer, and Al_2_O_3_ acts as a capping layer.

In summary, it is essential to verify that the Ta/CoFeB/Ir/MgO portion of the stack, which governs the magnetic properties and symmetry, is amorphous or polycrystalline.

**Crystalline orientation data to justify our symmetry-based argument**

To validate the symmetry of our system, we performed XRD measurements to confirm C∞v symmetry. The raw XRD data are shown in the inset of **Fig. S4a**. The bright spot corresponds to the crystalline Si substrate, while the three ring patterns arise from polycrystalline SiO_2_, Ta, and Al_2_O_3_. Their peak positions and intensities were extracted from the inset. No detectable diffraction signals were observed from the CoFeB, Ir, and MgO layers, indicating that they are amorphous. **Fig. S4b** shows the data with the Si-substrate signal removed, revealing the three rings more clearly.

In conclusion, the XRD analysis demonstrates that within the Ta/CoFeB/Ir/MgO stack, the CoFeB, Ir, and MgO layers are amorphous while Ta is polycrystalline, confirming that the overall stack possesses C∞v ​ symmetry.

| 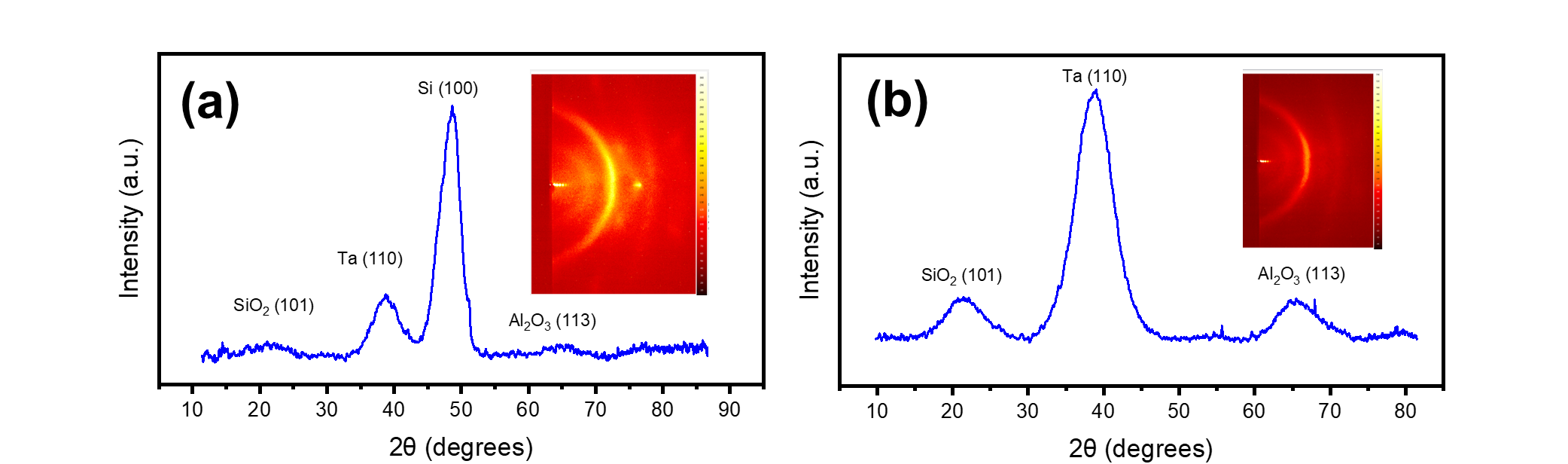 |
| --- |
| **Fig. S4 \| XRD measurements of the material stack. (a),** XRD data. Inset is the raw data. Bright spot: Si-substrate. Three ring shapes: SiO_2_, Ta, and Al_2_O_3_. **(b),** XRD data excluding the Si-substrate. |

**MOKE imaging data to justify our symmetry-based argument**

To further justify the C∞v symmetry of our Ta/CoFeB/Ir/MgO stack, we performed magnetic domain imaging across three representative samples. The first sample is our system of interest, while the second and third are control samples with intentionally introduced in-plane uniaxial anisotropy along the x- and y-directions, respectively.

As shown in **Fig. S5ai**, the domain pattern in our system exhibits labyrinthine stripe domains with no preferential in-plane orientation. The corresponding two-dimensional fast Fourier transform (FFT) pattern forms a nearly perfect circle in reciprocal space (**Fig. S5aii**), indicating isotropic in-plane behavior and supporting the presence of effective C∞v symmetry.

In contrast, the control sample with an additional uniaxial anisotropy along x-axis shows elongated stripe domains aligned in y-axis (**Fig. S5bi**), and its FFT reveals an elliptical pattern stretched along the x-direction (**Fig. S5bii**). Similarly, the sample with anisotropy along y-axis exhibits stripe domains elongated in the x-axis (**Fig. S5ci**), with the FFT compressed along y-axis (**Fig. S5cii**).

We note that deviations from circular symmetry in the FFT reflect any form of in-plane symmetry breaking—such as magnetic anisotropy, anisotropic DMI, strain-induced effects, or morphological features. Therefore, these comparisons highlight that only our system produces an isotropic FFT response, confirming that it lacks any directional bias—whether magnetic, interfacial, or structural—and can therefore be described by C∞v symmetry.

| 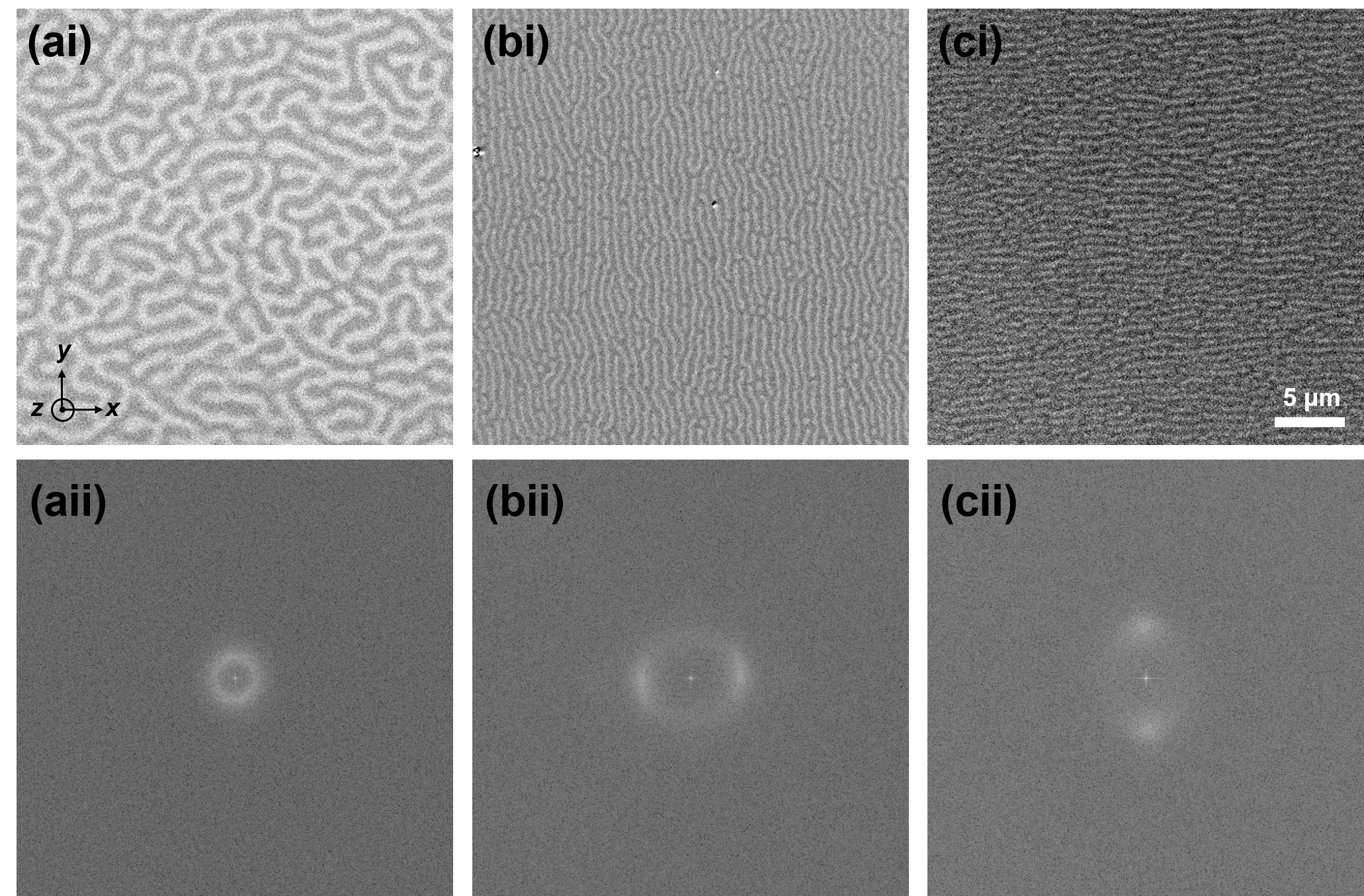 |
| --- |
| **Fig. S5 \| MOKE imaging and their FFT of our system and two controlled system. (ai),** MOKE image for our isotropic system. **(aii),** FFT result of **(ai)**. **(bi),** MOKE image for system with an additional x-axis anisotropy. **(bii),** FFT result of **(bi)**. **(ci),** MOKE image for system with an additional y-axis anisotropy. **(cii),** FFT result of **(ci)**. |

**Fig. S6.**

| **** |
| --- |
| **Fig. S6 \| Topological number of skyrmion and antiskyrmion. (a),** Spin texture of skyrmion and antiskyrmion. **(b),** Abstraction of spin textures. In-plane projection of skyrmion or antiskyrmion spin is depicted by the black arrows. Polarity $(p)$ is illustrated as the blue circle-with-cross. Vorticity $(\nu)$ is illustrated as the red circle-with-arrow. **(c),** Calculation of topological number. |

**Fig. S7.**

| **** |
| --- |
| **Fig. S7 \| Stable stripe domain state at zero field.** Stripe domains stay stable and no fragmentation event occurs. |

**Fig. S8.**

| **** |
| --- |
| **Fig. S8 \| Skyrmion number tracking procedure. (a),** Original MOKE images. **(b),** Thresholding and binarized images. **(c),** Skyrmion number tracking images. Skyrmion size is within the range of 50 – 200 pixels. Red numbers label the skyrmions. **(i)** corresponds to the first frame (time zero). **(ii)** No. 68 frame (at 34 s). **(iii)** No. 150 frame (at 75 s). |

**Fig. S9.**

| **** |
| --- |
| **Fig. S9 \| Equilibrium states of the skyrmion fragmentation events.** |

**Figs. S10 & S11**

It is important to demonstrate how closely the simulations reproduce the experimental results. To this end, we have performed additional simulations using parameters that best match the experiment in order to quantitatively validate our findings.

As shown in **Fig. S10**, the main discrepancy is that the simulated skyrmion size does not exactly match the experimental value. This is because, in our original simulations, we used a relatively large DMI value of 0.003 J/m² to reduce computational cost. A larger DMI leads to smaller domain and skyrmion sizes, which shortens simulation time. All other parameters were selected to match our experimental data.

To improve agreement with experiment, we now perform additional simulations with more realistic DMI values of 0.0025 J/m², 0.002 J/m², 0.0015 J/m², and 0.001 J/m². The results show that decreasing the DMI systematically increases the domain and skyrmion sizes (**Fig. S10**).

In all cases, we still observe the antiskyrmion-mediated, thermally driven stripe-to-skyrmion transition at a fixed field, confirming the robustness of the effect.

| 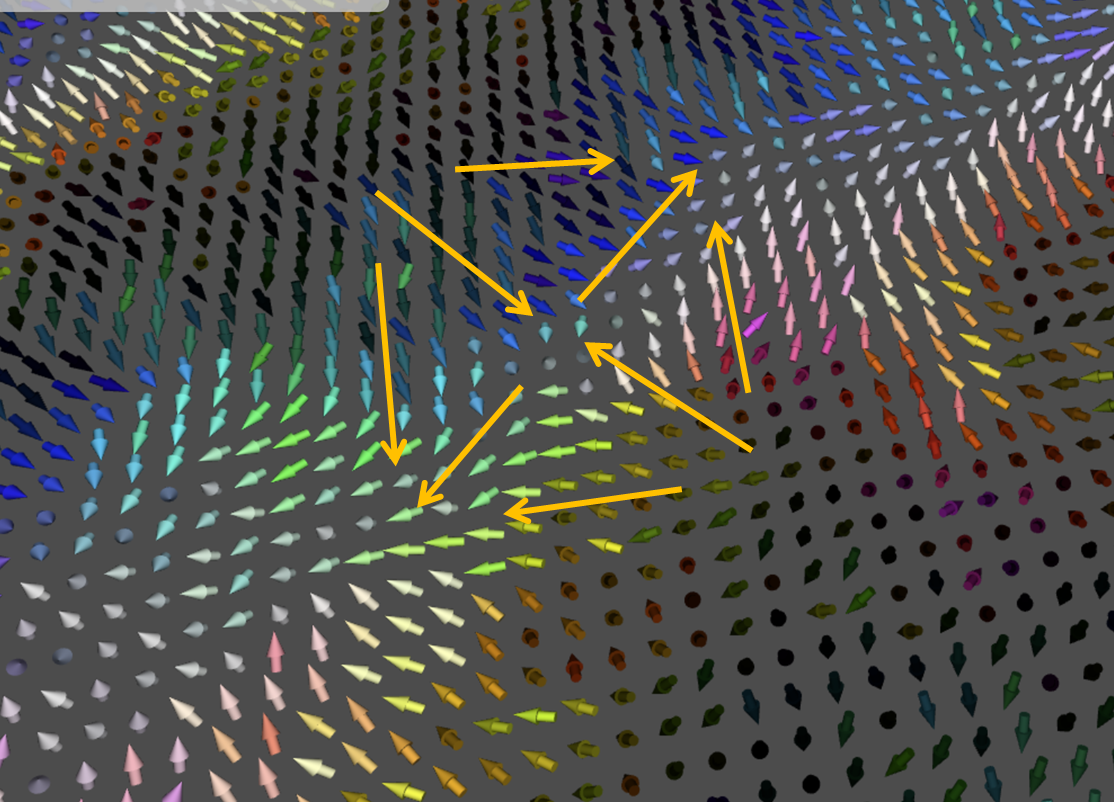 |
| --- |
| **Fig. S10 \| Simulation to quantitatively justify the experiment.** Simulation with various DMI value (0.003, 0.0025, 0.002, 0.0015, 0.001) J/m^2^ in an $800\times800\times1 {nm}^{3}$ simulation box. With decreasing DMI values, the feature size of skyrmions is increasing. **Right figure:** Observed antiskyrmion spin texture during this transition. |

We further extract the skyrmion size as a function of DMI from these simulations and plot the results in **Fig. S11a**, with the final data point corresponding to the experimental value (0.1 mJ/m²). Extrapolating the simulation curve indicates consistency with the experimental skyrmion size when the DMI is close to the experimental value, thereby validating our approach. However, a full-scale simulation with a 50 μm × 50 μm area would be required to completely replicate the experiment, which is beyond our current computational capability. This is why we chose a larger DMI value in the simulations.

In addition, to reproduce the experimental observation of an exponential increase in skyrmion number and the formation of fewer skyrmions from stripes at lower fields (**Fig. 3b**, Main Text), we performed new simulations under these conditions. The results (**Fig. S11b**) show the same trend as observed experimentally.

In summary, despite computational constraints, our simulations reproduce the core experimental phenomena and support the robustness of our conclusions.

| 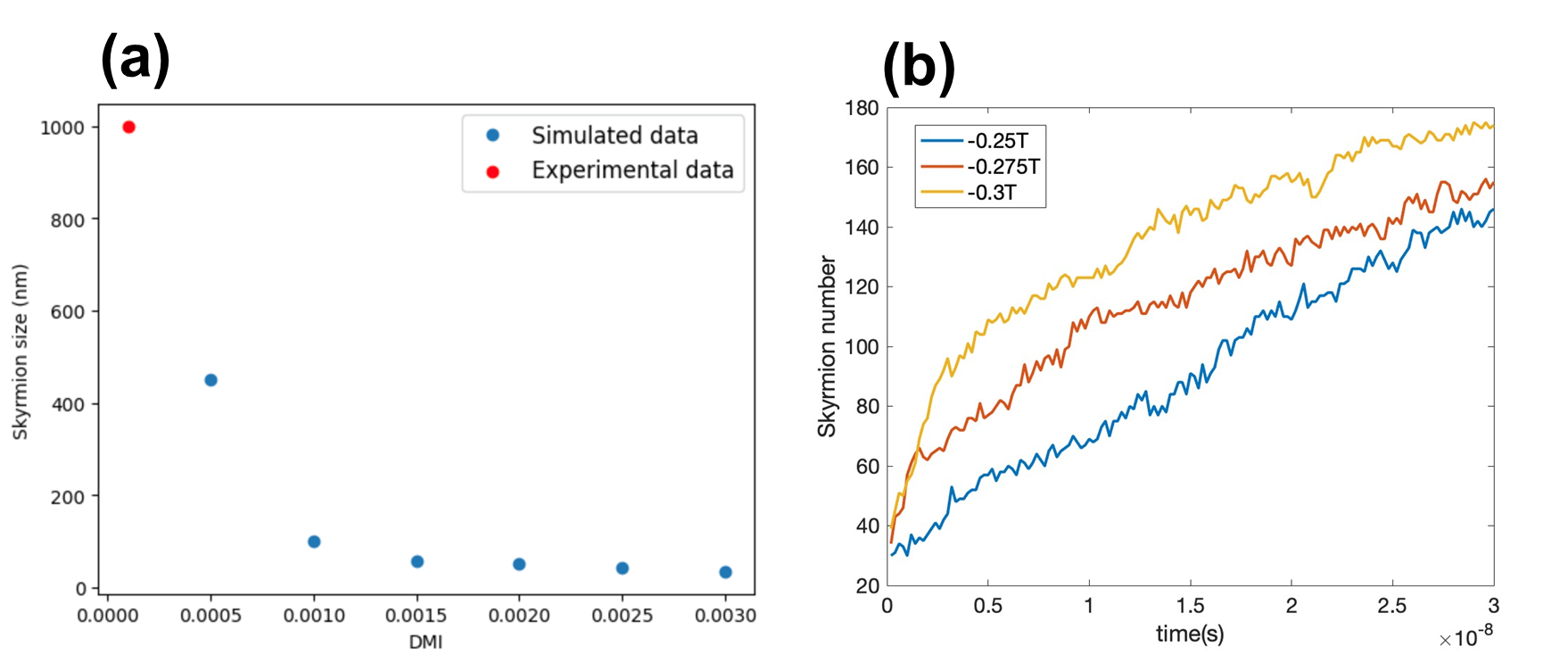 |
| --- |
| **Fig. S11 \| (a),** Skyrmion size vs. DMI value. **(b),** Stripe to skyrmion transformation at different fields. |

**Fig. S12.**

| 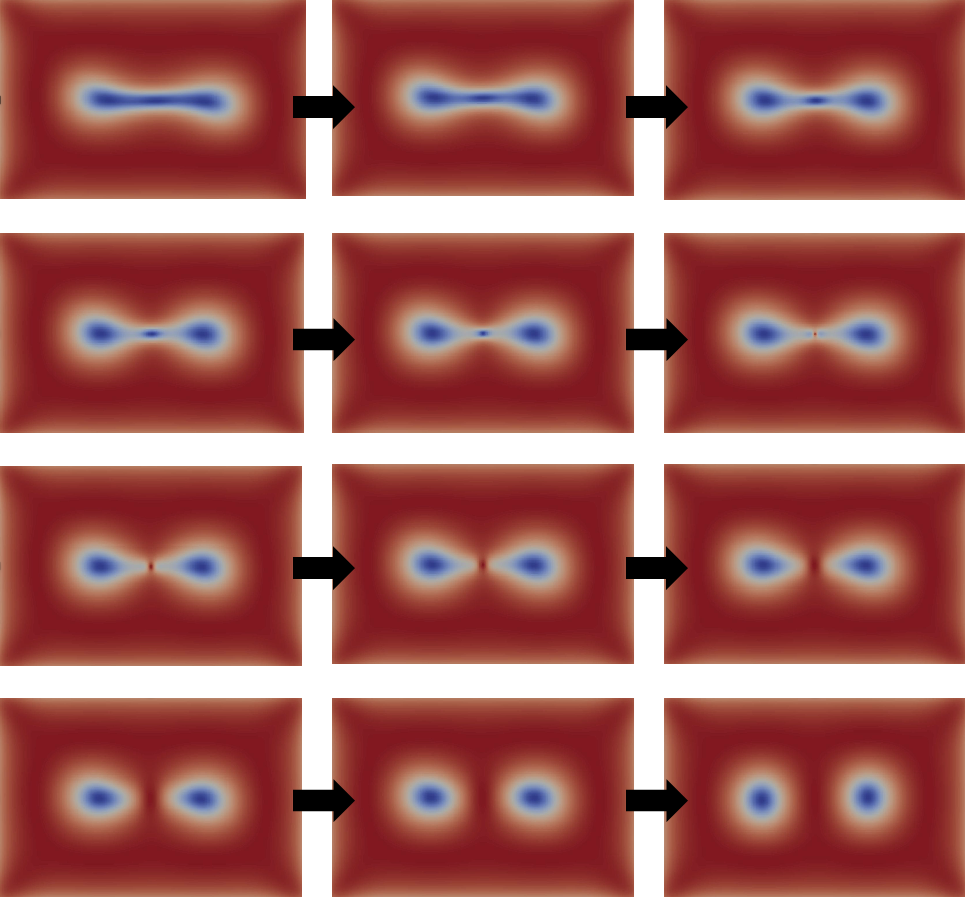 |
| --- |
| **Fig. S12 \| Representative states on the reaction coordinate (minimum energy path).** Single-stripe-domain transforms into two-skyrmion-state following the minimum energy path. 12 representative states are shown following the sequence of energy evolution. The simulation box is $400\times200\times1 {nm}^{3}$. |

**Figs. S13 & S14**

**Symmetry-breaking mechanism of antiskyrmion formation**

The antiskyrmion is typically stabilized by anisotropic DMI, whereas our system has isotropic DMI. For an antiskyrmion to form under such conditions, symmetry breaking is required.

In our multilayer, the energy barrier is very low—only ~3 Oe is needed to fully saturate the film, as shown by the hysteresis loop in **Fig. 2a**. Consequently, the energy barriers between different magnetic states are also small, and thermal fluctuations are sufficient to drive the transformation of stripe domains into skyrmions. During this transition, an antiskyrmion can transiently emerge as a metastable state. However, because an antiskyrmion requires anisotropic DMI for stabilization, it remains unstable in our isotropic DMI system and is quickly annihilated by thermal fluctuations. Thus, the observed antiskyrmion is a transient state, and the effective symmetry breaking is provided by thermal fluctuations in a low–energy-barrier system.

**Section 2. Mechanism and process of antiskyrmion formation**

The key question is why an antiskyrmion forms during the stripe-to-skyrmion transition and how this can occur in a system with isotropic DMI.

We address this through an energy minimization analysis. Experimentally, the initial state is a stripe-domain configuration and the final state is a two-skyrmion configuration. To connect these states, we calculated the minimum energy path for the transition (details provided in Methods). The result, shown in **Fig. S13a** (reproduced **Fig. 3d** of the Main Text), reveals that during the transition from State ① (stripe domain) to State ⑤ (two skyrmions), an intermediate antiskyrmion configuration appears at State ③. The inset with colored arrows illustrates the spin texture of State ③, which corresponds to an antiskyrmion.

| 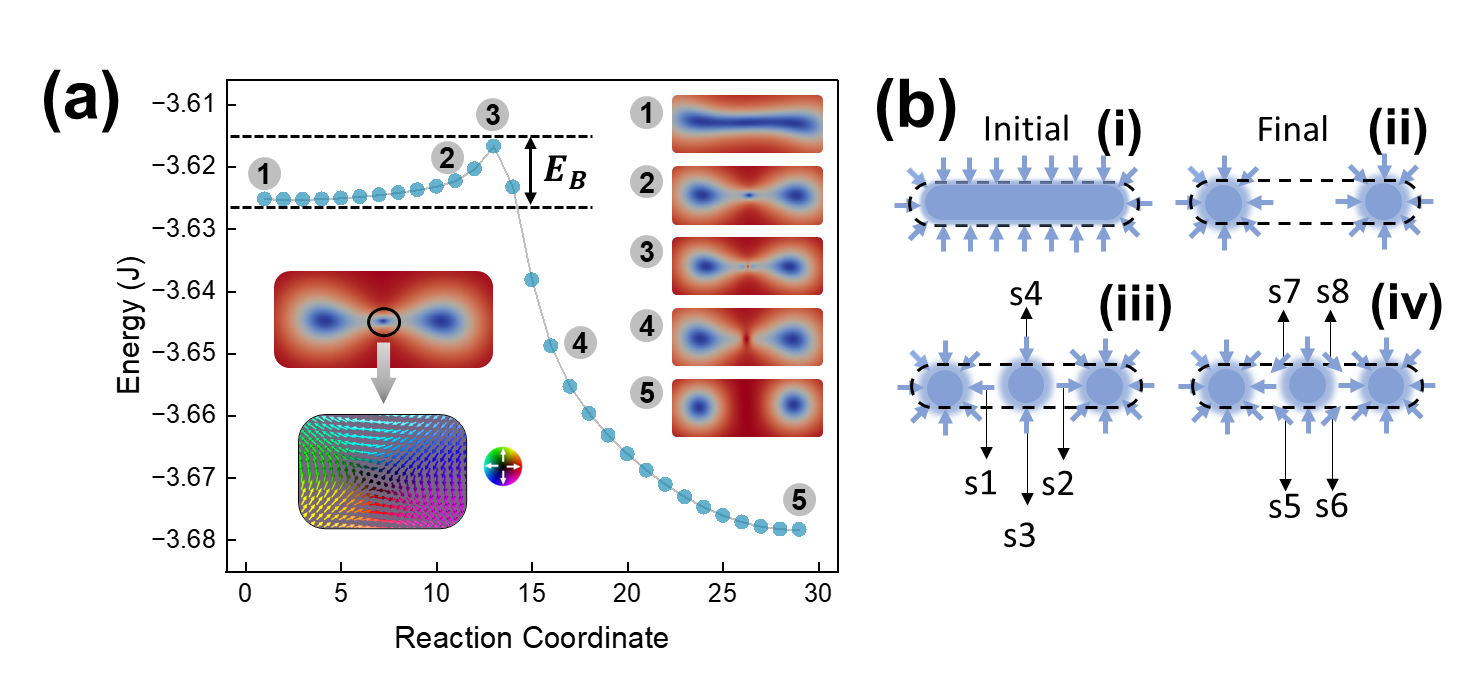 |
| --- |
| **Fig. S13 \| Minimum energy path calculation and the emergence of antiskyrmion. (a),** The calculated minimum energy path. **(b),** Illustration to explain the emergence of antiskyrmion. |

The emergence of the antiskyrmion facilitates a smooth transition and minimizes the energy. In other words, the antiskyrmion spontaneously appears as an intermediate state during the spin-texture evolution.

With isotropic DMI, the initial single-stripe state produces a Néel-type wall (**Fig. S13bi**), while the final configuration consists of two Néel-type skyrmions (**Fig. S13bii**). In the intermediate stage, the stripe core and two skyrmions coexist (**Fig. S13biii**). Here, the two Néel skyrmions define spin directions s1​ and s2​, while the Néel wall defines s3​ and s4​.

To minimize exchange energy, the spins must rotate smoothly from s4→s1 and s4→s2 ​, as well as from s3→s1 ​ and s3→s2 ​. This process generates intermediate spins s5, s6, s7, and s8, which collectively form an antiskyrmion in the center (**Fig. S13biv**). The role of exchange-energy minimization is confirmed by the minimum energy path calculation, shown in **Fig. S14a**.

In summary, isotropic DMI produces Néel-type spin textures, and during the stripe-to-skyrmion transition the energetically smoothest path is through an intermediate antiskyrmion at the center. However, since antiskyrmions require anisotropic DMI for stability, this state is unstable and is eventually annihilated by thermal fluctuations, resulting in the final two-skyrmion state.

The direction-resolved spin textures along the calculated minimum energy path are shown in **Fig. S14b**, where we observe the initial single-stripe state (State ①), the final two-skyrmion state (State ⑧), and the transient antiskyrmion emerging at an intermediate state (State ④).

| 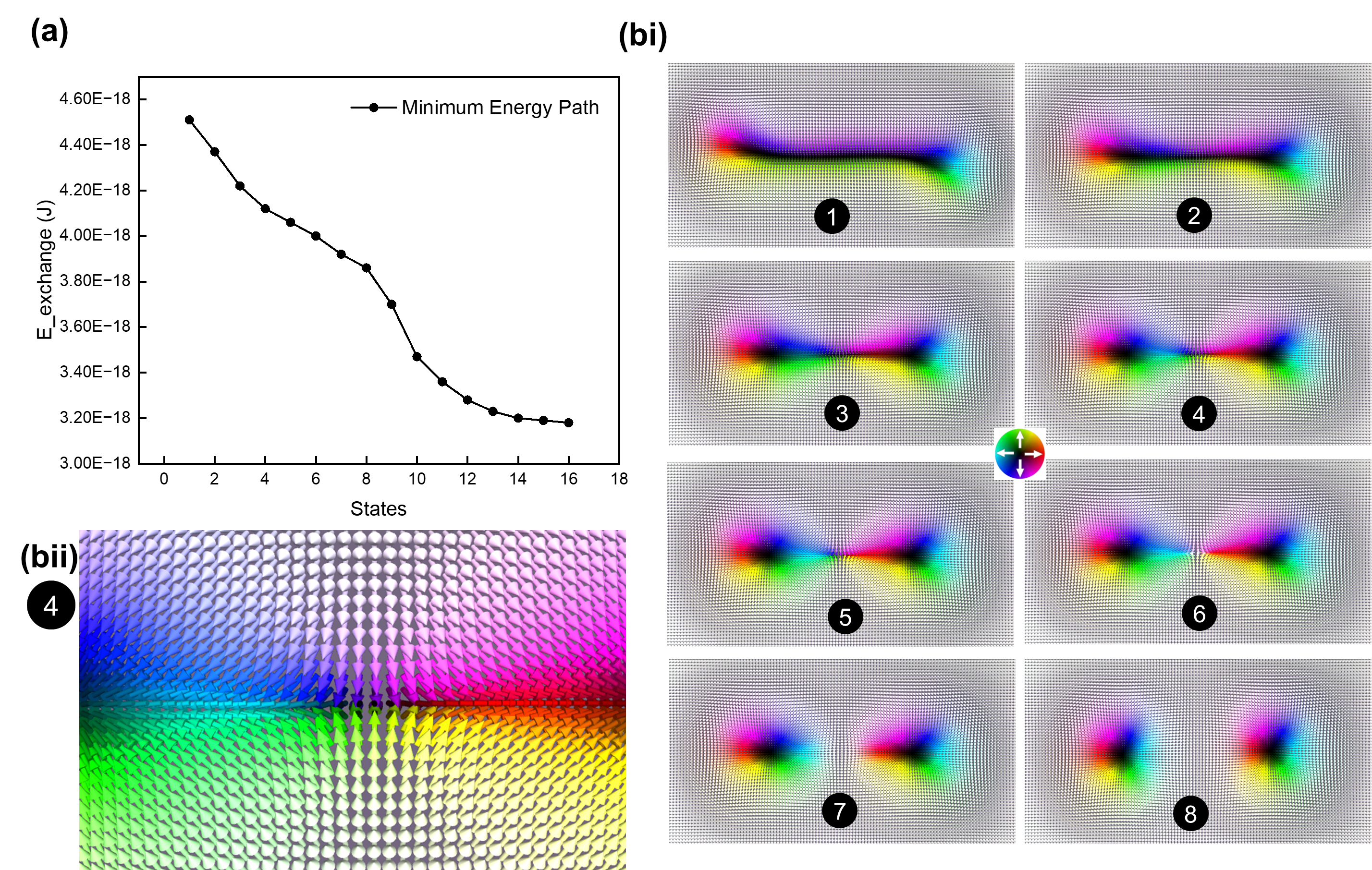 |
| --- |
| **Fig. S14 \| Minimum energy path calculation results. (a),** The minimization of exchange energy. **(bi),** State ①: Stripe domain, State ④: Antiskyrmion, State ⑧: two-skyrmion. White: +z, black: -Z, and in-plane direction is shown by the color wheel in the center. **(bii),** Magnified picture of State ④ to better resolve the antiskyrmion spin texture. |

**Figs. S15 & S16**

As discussed in the Main Text, the PMA directly controls the transition energy barrier between the single-stripe and two-skyrmion states, it also influences the stability and lifetime of the intermediate antiskyrmion state.

**Relationship between Ir thickness (PMA) and energy barrier**

To investigate the relationship between PMA and the transition energy barrier, we employed the GNEB method to calculate the minimum energy path between the two-skyrmion state and the single-stripe state. The calculation is performed using world size of $200\times200\times1$ cells with cell size of $2\times2\times1 {nm}^{3}$ and the following material parameters: exchange constant $1.3\times{10}^{-11} J/m$, saturation magnetization ${8.6\times10}^{5} A/m$, interfacial DMI ${1.3\times10}^{-3} J/m^{2}$, out-of-plane magnetic field $0.0754 T$.

The above parameters are fixed, we only vary the PMA energies: ${K_{u1}=4\times10}^{5} J/m^{3}$, ${K_{u2}=4.6\times10}^{5} J/m^{3}$, ${K_{u3}=5\times10}^{5} J/m^{3}$. The results are shown in **Fig. S15**. We observe the transient antiskyrmion state in all three cases (see lower panel of **Fig. S15**), which validates the calculation. The energy barrier *E_B_* increases with as the PMA increases (**Fig. S15** top panel).

| 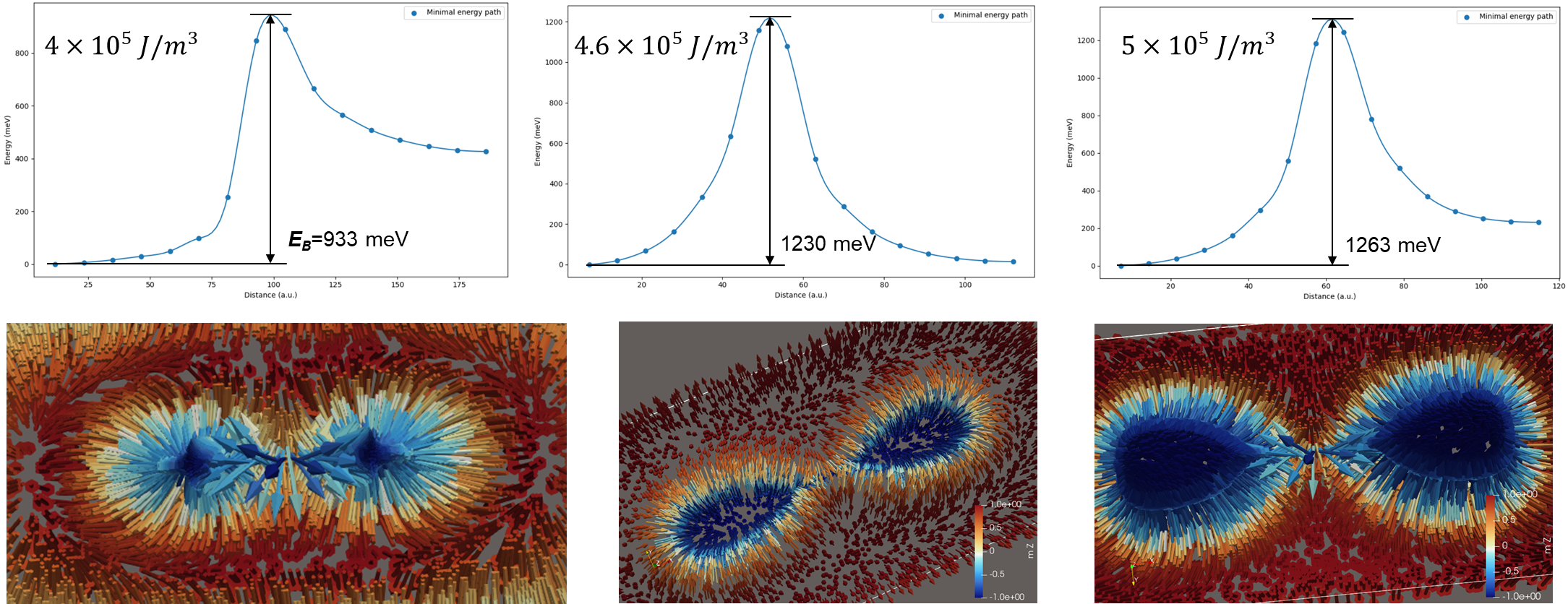 |
| --- |
| **Fig. S15 \| Relation between Ir thickness (PMA) and energy barrier. Top panel:** Minimum energy path for the transition from single-stripe to two-skyrmion state. **Lower panel:** Observed transient antiskyrmion state for these three PMA cases. |

**Relationship between Ir thickness (PMA), energy barrier, and antiskyrmion lifetime.**

The transient antiskyrmion emerges during the stripe-to-skyrmion transition. Since this process is thermally activated, the increase in skyrmion number follows an exponential dependence. The lifetime of the transient state can be extracted by fitting the skyrmion number evolution curve obtained from simulations, as shown in **Fig. S16**. (*Additional details of the fitting procedure and model are provided at the end of this section.*)

The extracted lifetimes are $5.09 ns$, $6.51 ns$, $6.84 ns$ for ${K_{u1}=3\times10}^{5} J/m^{3}$, ${K_{u2}=4\times10}^{5} J/m^{3}$, ${K_{u3}=5\times10}^{5} J/m^{3}$, respectively. We can see higher PMA gives rise to longer antiskyrmion lifetime. Furthermore, by fitting the simulation of **Fig. 3c** of the Main Text, we get a lifetime of $9.17 ns$.

In summary, we conclude that higher PMA gives rise to higher energy barrier, and higher energy barrier leads to longer antiskyrmion lifetime.

|  |
| --- |
| **Fig. S16 \| Relation between Ir thickness (PMA) and antiskyrmion lifetime.** The fitted lifetimes are 5.09ns. 6.51ns, 6.84ns for Ku1,2,3, respectively. |

**Details of the fitting and model:**

Given the assumption that a transient antiskyrmion has a constant transition rate *Γ*, we construct the following model. Initially, the system consists of a stripe domain with length *N*, which can finally fragment into *N* individual skyrmions, each with unit size. Fragmentation can occur at any position along the stripe. In total, *N-1* fragmentation events occur, which converts the stripe domain to *N* skyrmions. At each fragmentation step, a transient antiskyrmion mediates the process with the constant transition rate *Γ*. At any given time *t*, the system exists as a mixture of stripe domains and skyrmions formed from prior transition events. The length of stripe domain survival portion *S(t)* at given time t obeys

$$\frac{dS}{dt}=-\Gamma S$$

Physically, this implies that longer stripe domains fragment into skyrmion more frequently, as there are more potential positions for fragmentation to occur. As a result, the overall transition rate decreases with the shortening length of the remaining stripe. This leads to a dynamic evolution governed by a time-dependent rate equation, which can be solved as:

$$S\left( t \right)=e^{-t/\tau}, \tau=\frac{1}{\Gamma}$$

Then the total fragmented skyrmions after duration *t* follows such distribution:

$$F\left( t \right)=N-{Ne}^{-t/\tau}$$

Transient antiskyrmions typically annihilate or transform by thermally activation over an energy barrier. Such equation characterizes the standard kinetics for transient antiskyrmions in a steady environment—and *τ* returned by the fit is the mean antiskyrmion lifetime. It’s the mathematical solution of a constant-rate fragmentation process.

In real data analysis, the skyrmion number are fitted with the following equation.

$$F\left( t \right)=a+be^{-(t-c)/\tau}$$

Here *a, b, c* are fitting coefficients. $\tau$ is the antiskyrmion mean lifetime, which can be extracted by fitting the skyrmion number evolution curve from simulation.

**Fig. S17.**

| **** |
| --- |
| **Fig. S17 \| Net magnetization evolution during the fragmentation phenomena. (a)(i),** $Q=+1$ stripes transition into skyrmions, leading to the annihilation of $+z$ core spin antiskyrmions. **(a)(ii),** $Q=-1$ stripes transition into skyrmions, leading to the annihilation of $-z$ core spin antiskyrmions. **(b),** Evolution of the net magnetization in $x,y,z-$direction ($M_{x,y,z}$) for these two cases. |

**Fig. S18.**

We fabricate devices with different dimensions; the size of the center confinement region is defined by the width of the horizontal and vertical crossbars. As a result, the **Fig. S18(a)** device has a dimension of roughly 4 μm by 4 μm, **(b)** device 6 μm by 6 μm, **(c)** device 8 μm by 8 μm, **(d)** device 10 μm by 10 μm, and **(e)** device 30 μm by 30 μm. We can see that fewer and fewer skyrmions are allowed to exist in the center region as the size goes down, demonstrating the confinement effect. Finally, only two skyrmions are presented in the 4 μm by 4 μm device, same dimension and similar observation as the device we presented in **Fig. 4c** of the Main Text. In summary, by gradually shrinking the device size, the confinement effect is demonstrated.

| 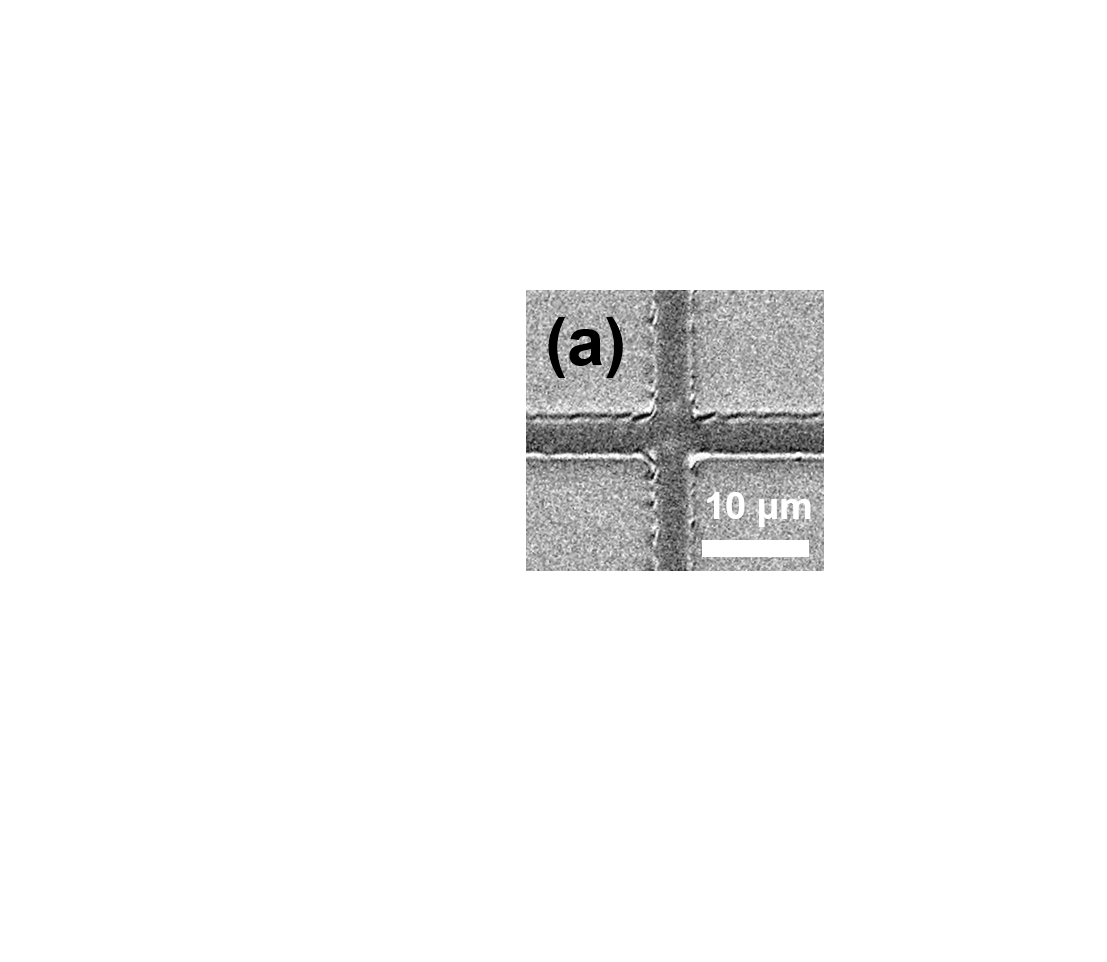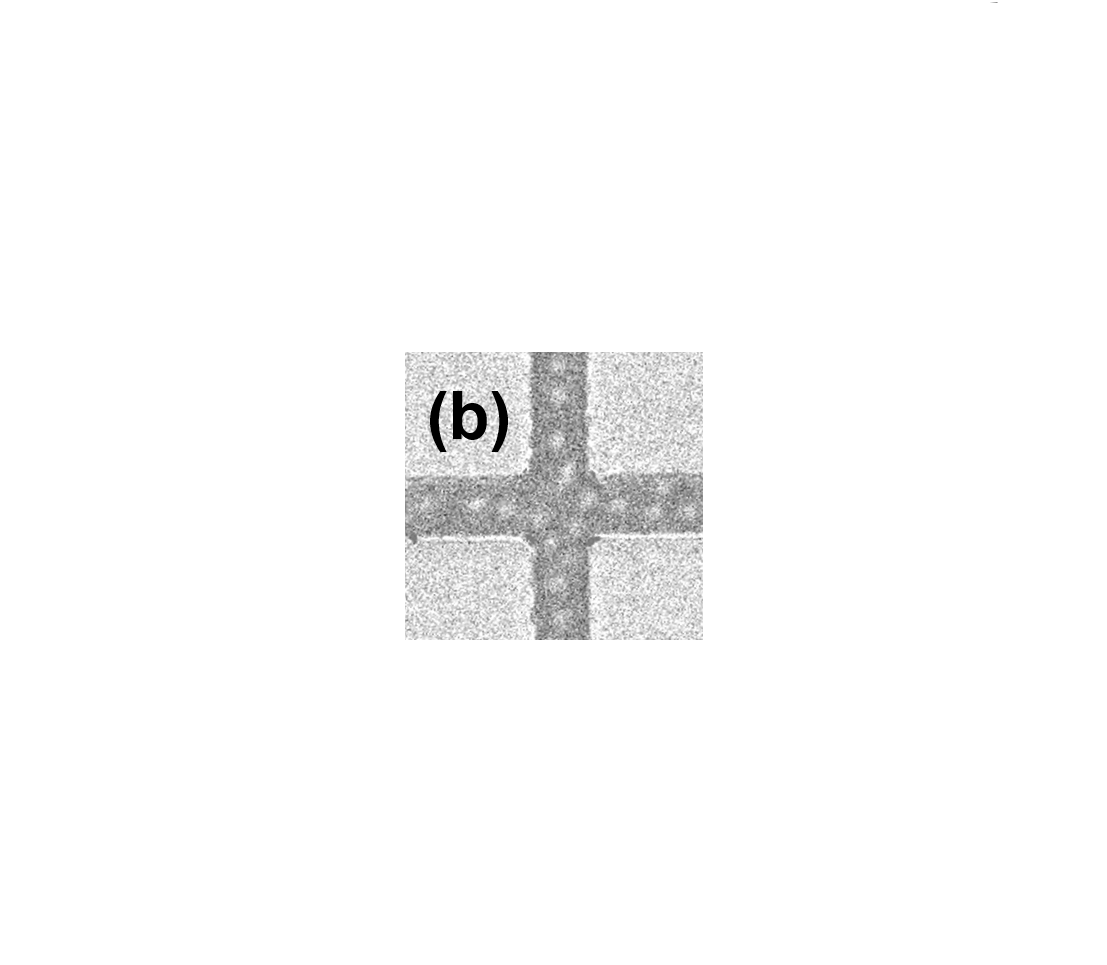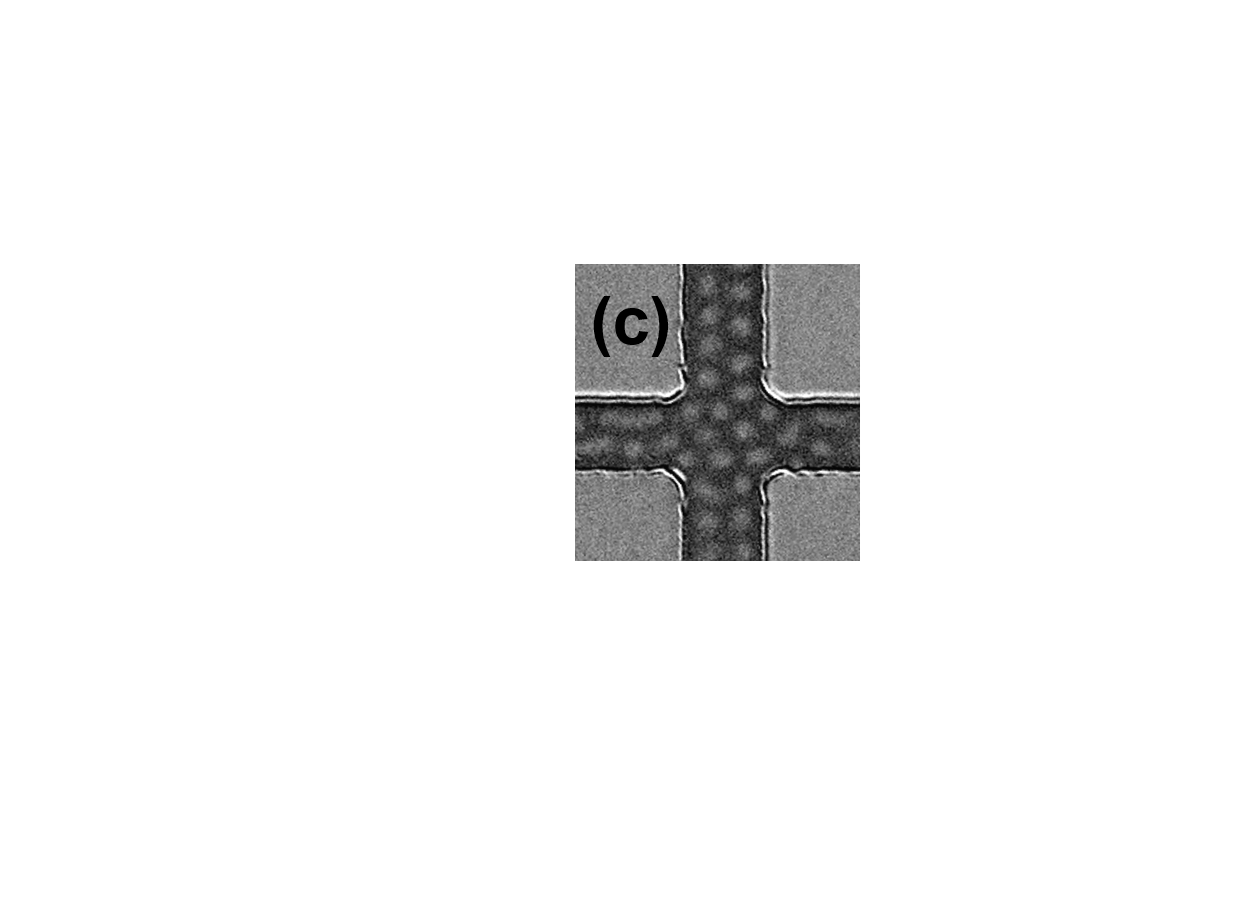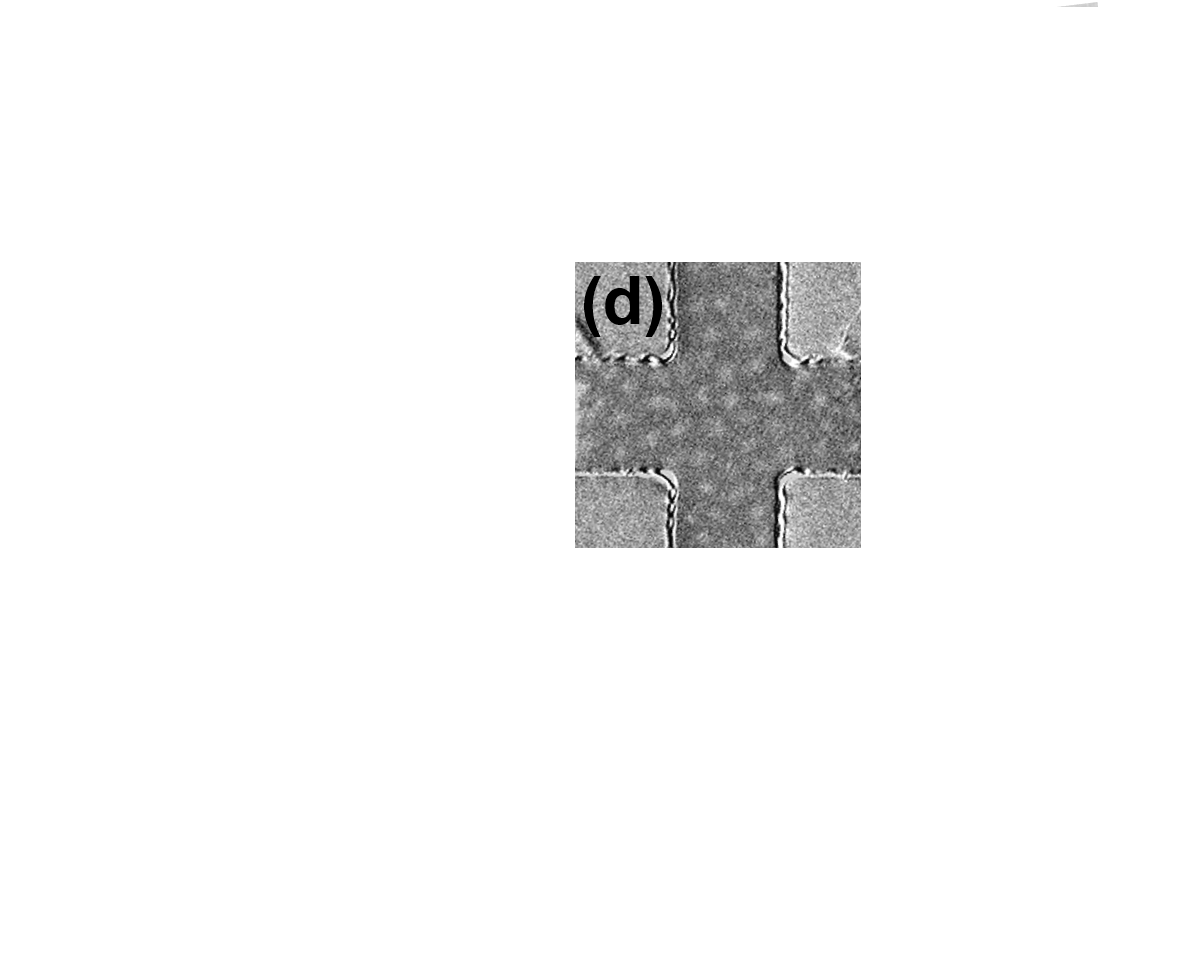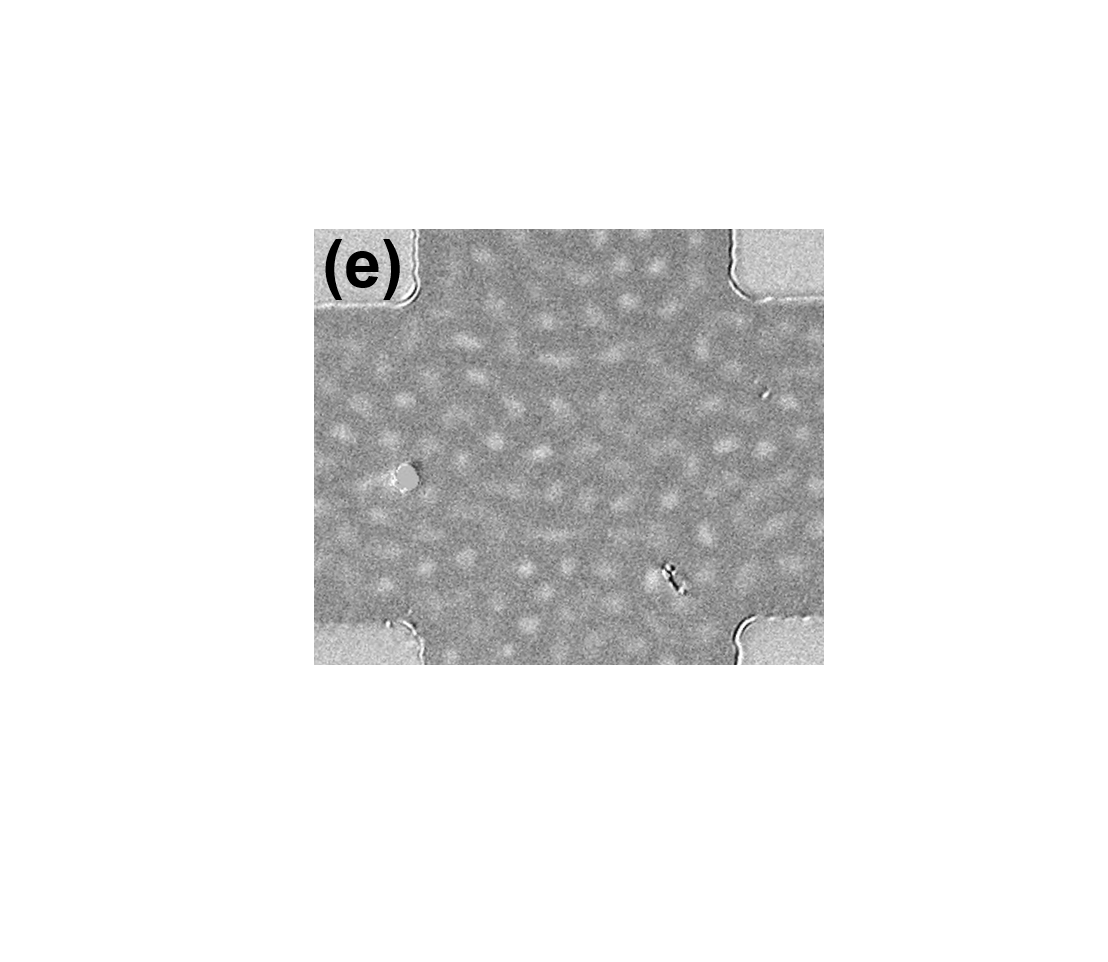 |
| --- |
| **Fig. S18 \| MOKE images of confinement devices with different dimensions. (a),** The 4 μm device. **(b),** The 6 μm device. **(c),** The 8 μm device. **(d),** The 10 μm device. **(e),** The 30 μm device. Fewer and fewer skyrmions are allowed to exist in the center region as the size goes down, showing the confinement effect. |

**Fig. S19.**

| **** |
| --- |
| **Fig. S19 \| Simulation of single antiskyrmion annihilation under geometric confinement.** Left figure: Time evolution of $M_{z}$ (left $y-$axis) and $Q_{Skyrmion}$ (right $y-$axis). Right figure: Magnetic states before and after the fragmentation. The initial state corresponds to a single stripe domain ($Q=+1$), and the final state shows two skyrmions ($Q=+2$) following the annihilation of the confined antiskyrmion. |

**Fig. S20.**

| **** |
| --- |
| **Fig. S20 \| Calibration of the field ramp-up and stabilization time.** The blue-shaded rectangle denotes the transition region. The field ramp up and stabilization time is $18.8 ms$. $V_{AHE}=-295.8 mV$ at positive $M_{z}$ saturation, and $V_{AHE}=11 mV$ at negative $M_{z}$ saturation. |

**Fig. S21.**

| **** |
| --- |
| **Fig. S21 \| Raw data for stochastic bit generation.** A representative example consisting of 974 data points is shown. To sample the stochastic bit stream, a $5 ms$ sampling interval is used. The signal fluctuates between the two blue dashed lines and around the average value indicated by the red line. Bits ‘1’ and ‘0’ are assigned based on whether the signal is above or below the average $V_{AHE}$ (red line). The resulting bit stream is presented in Fig. 4f of the main text. |

**Figs. S22 & S23.**

**Measurements on circular confinement device**

To investigate the effects of device confinement geometry and shape anisotropy, we fabricated a circular confinement device, which differs only in shape from the square device shown in **Fig. 4c** of the manuscript. We repeated the same measurements performed on the square device.

The results are summarized in **Fig. S22**: the MOKE images (**Fig. S22a**) show a single confined stripe domain (white stripe) transitioning into a two-skyrmion configuration (two white circular objects). The corresponding dynamics measured by AHE are shown in **Fig. S22b**. We further collected a 1-million-bit random bitstream, with a representative 200-bit segment shown **in Fig. S22c**. The NIST randomness test results (**Fig. S22d**) demonstrate that the generated sequence exhibits the same intrinsic bias (~0.5) and passes all 15 statistical tests, consistent with the square device.

For comparison, the results of the square device are displayed in the bottom row of **Fig. S22** (reproduced form **Fig. 4** of the Main Text). In both cases, the MOKE images confirm the same antiskyrmion-mediated stripe-to-skyrmion transition, the AHE oscillation amplitude and period confirm similar antiskyrmion dynamics, and the NIST test confirms similar energy barriers for random number generation. These results indicate that shape anisotropy does not play a significant role in the observed phenomena.

| 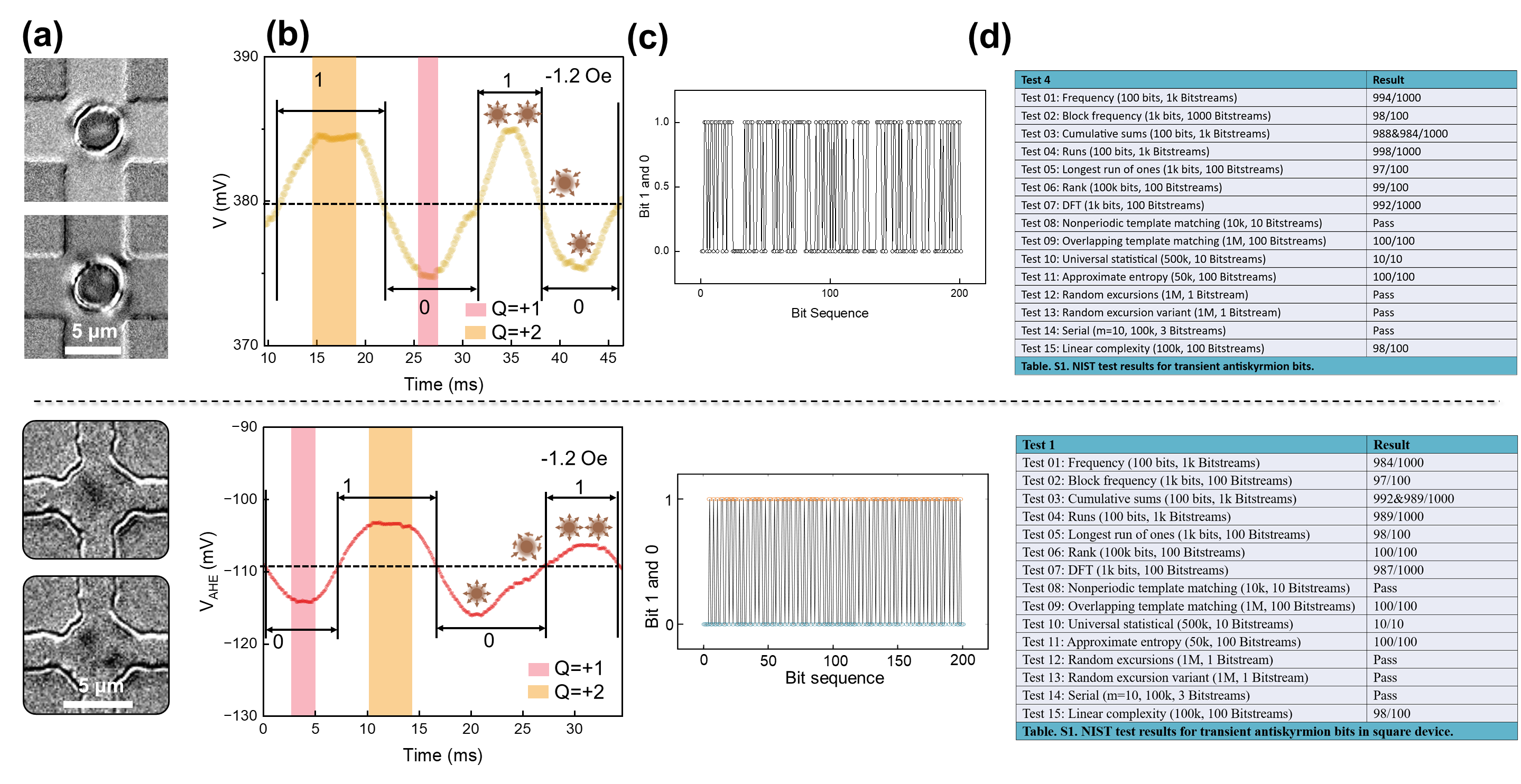 |
| --- |
| **Fig. S22 \| Measurements on circular confinement device. (a),** MOKE images of single-stripe to two-skyrmion transition. **(b),** Dynamics measurement by AHE using the same measurement condition as in Fig. 4 of the manuscript. **(c),** 1 million random numbers are collected from this circular confinement device, 200 representative bit-stream is shown here. **(d),** NIST true random number test result for these 1 million random numbers. The results of the square device are arranged at the bottom row for comparison. |

**Simulation of circular and triangular confinement device**

To further corroborate this claim, we performed additional simulations using circular and triangular confinement geometries. As shown in **Fig. S23**, the results are consistent: an antiskyrmion appears during the fragmentation of a stripe domain into two skyrmions, matching the behavior observed in the square device presented in the manuscript.

In ultrathin films (1 nm), the effective shape anisotropy is primarily governed by the film thickness (out-of-plane demagnetization field) rather than by the lateral confinement geometry of the device. As a result, the device shape does not play a significant role in determining the property of the transient antiskyrmion. In our case, the dominant factors controlling the evolution of the transient state are the thermal fluctuations and PMA, which outweigh possible contribution from lateral shape effects.

| 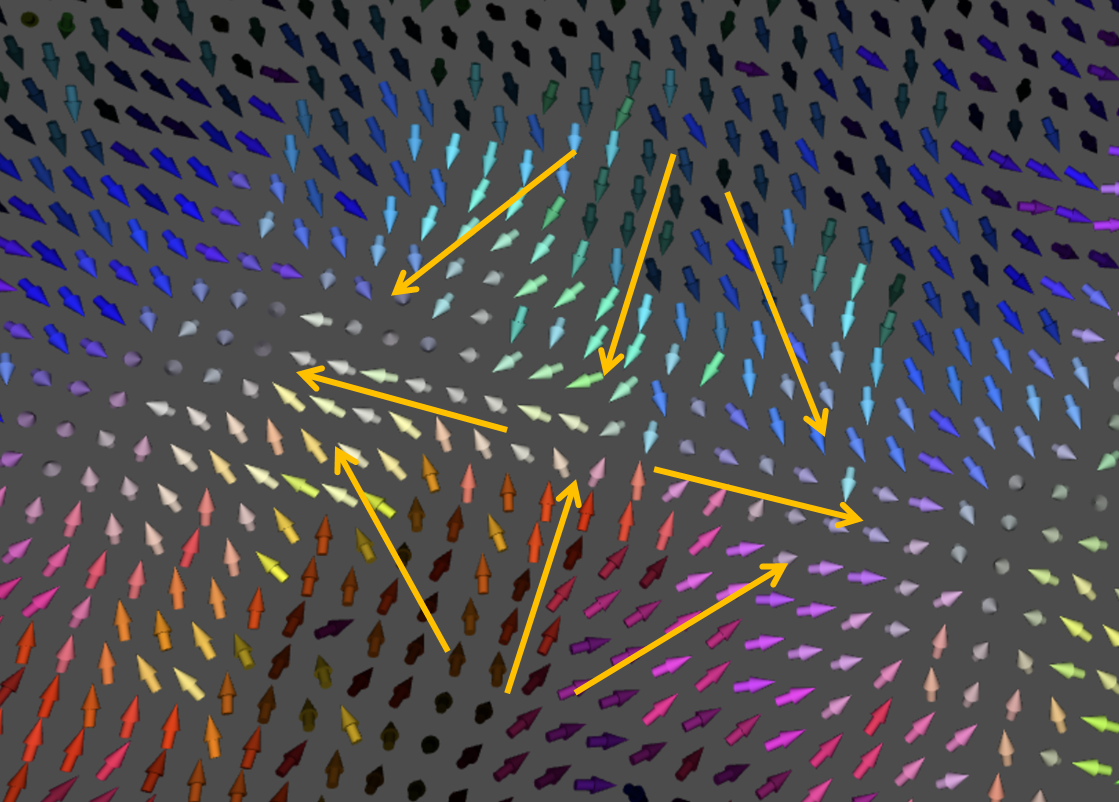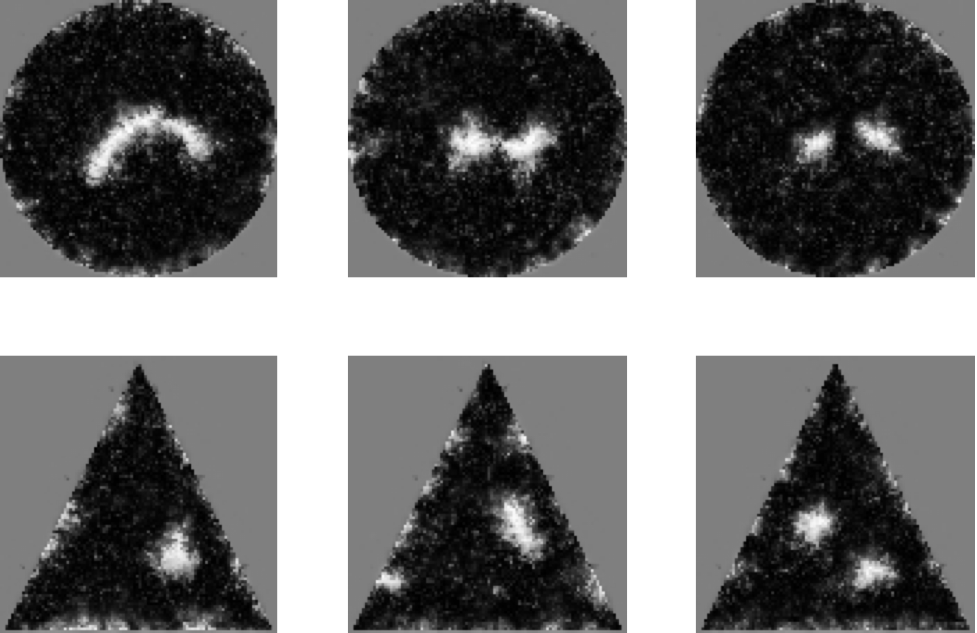 |
| --- |
| **Fig. S23 \| Micromagnetic simulation of confinement devices with different shapes. Left figures:** Skyrmion fragmentation in different shape confinement. **Right figure:** Antiskyrmion observed during this process. |

**Fig. S24.**

**Tuning probability at the device level**

As discussed in the Main Text, the energy barrier—and thus the transition probability—can be controlled by the applied magnetic field. To demonstrate this effect, we applied two different bias fields (1.1 Oe and 1.3 Oe) and measured the probability, defined as the ratio of zeros and ones. The mechanism is illustrated in **Fig. S24a**. For each condition, we generated 1 million random numbers, with representative 200-bit streams shown in **Figs. S24bi** and **S24ci**. At 1.3 Oe, the ratio of 0/1 is 60/40, favoring the 0-state (two-skyrmion state), whereas at 1.1 Oe, the ratio of 0/1 is 30/70, favoring the 1-state (one-stripe state). Both cases successfully pass the NIST randomness tests (**Figs. S24bii** and **S24cii**). Together with the 1.2 Oe case presented in **Fig. 4f** of the manuscript (50/50 ratio), these results demonstrate that the probability distribution can be tuned by the bias field.

Beyond bias-field tuning, voltage-controlled magnetic anisotropy (VCMA) could be implemented to enable more advanced CMOS integration, offering greater scalability and energy efficiency.

| 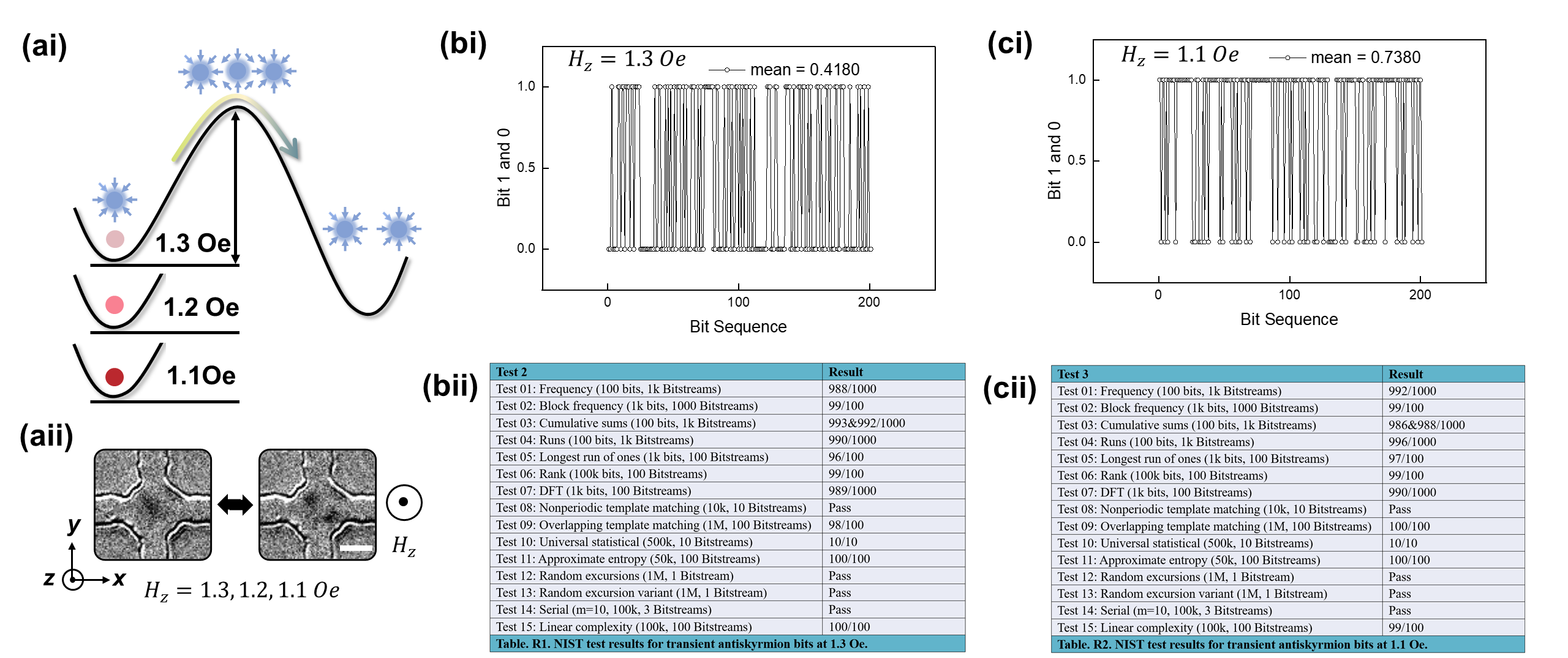 |
| --- |
| **Fig. S24 \| Tunable probability by adjusting the bias field. (ai),** The energy barrier is fine-tuned by the bias field, resulting in tunable transition probability. **(aii),** Measurement setup. 1.3 Oe, 1.2 Oe, and 1.1 Oe field is applied. **(bi),** 200-point bit-stream for the 1.3 Oe case. **(bii), N**IST random number test results of 1.3 Oe case. **(ci),** 200-point bit-stream for the 1.1 Oe case. **(cii),** NIST random number test results of 1.1 Oe case. |

**Fig. S25.**

|  |
| --- |
| **Fig. S25 \| Energy landscape in terms of skyrmion number.** The energy minimum of 2D Heisenberg model with exchange interaction only is quantized in skyrmion number. |

**Fig. S26.**

|  |
| --- |
| **Fig. S26 \| Block flow diagram for solving the molecular (protein) docking problem.** |

**Table. S1.**

| **Test** | **Result** |
| --- | --- |
| Test 01: Frequency (100 bits, 1k Bitstreams) | 984/1000 |
| Test 02: Block frequency (1k bits, 100 Bitstreams) | 97/100 |
| Test 03: Cumulative sums (100 bits, 1k Bitstreams) | 992&989/1000 |
| Test 04: Runs (100 bits, 1k Bitstreams) | 989/1000 |
| Test 05: Longest run of ones (1k bits, 100 Bitstreams) | 98/100 |
| Test 06: Rank (100k bits, 100 Bitstreams) | 100/100 |
| Test 07: DFT (1k bits, 100 Bitstreams) | 987/1000 |
| Test 08: Nonperiodic template matching (10k, 10 Bitstreams) | Pass |
| Test 09: Overlapping template matching (1M, 100 Bitstreams) | 100/100 |
| Test 10: Universal statistical (500k, 10 Bitstreams) | 10/10 |
| Test 11: Approximate entropy (50k, 100 Bitstreams) | 100/100 |
| Test 12: Random excursions (1M, 1 Bitstream) | Pass |
| Test 13: Random excursion variant (1M, 1 Bitstream) | Pass |
| Test 14: Serial (m=10, 100k, 3 Bitstreams) | Pass |
| Test 15: Linear complexity (100k, 100 Bitstreams) | 98/100 |
| **Table. S1 \| NIST test results for transient antiskyrmion bits.** | |
